# Demographic and Clinical Characteristics Associated With the Failure of Nonoperative Management of Uncomplicated Appendicitis in Children: Secondary Analysis of a Nonrandomized Clinical Trial

**DOI:** 10.1001/jamanetworkopen.2022.9712

**Published:** 2022-05-02

**Authors:** Peter C. Minneci, Erinn M. Hade, Lindsay A. Gil, Gregory A. Metzger, Jacqueline M. Saito, Grace Z. Mak, Ronald B. Hirschl, Samir Gadepalli, Michael A. Helmrath, Charles M. Leys, Thomas T. Sato, Dave R. Lal, Matthew P. Landman, Rashmi Kabre, Mary E. Fallat, Jennifer N. Cooper, Katherine J. Deans

**Affiliations:** 1 Center for Surgical Outcomes Research, Abigail Wexner Research Institute at Nationwide Children’s Hospital, The Ohio State University College of Medicine, Columbus; 2 Department of Pediatric Surgery, Nationwide Children’s Hospital, Columbus, Ohio; 3 Department of Population Health, Division of Biostatistics, New York University Grossman School of Medicine, New York; 4 Division of Pediatric Surgery, Department of Surgery, Washington University School of Medicine, St Louis, Missouri; 5 Section of Pediatric Surgery, Department of Surgery, University of Chicago Medicine and Biologic Sciences, Chicago, Illinois; 6 Division of Pediatric Surgery, Department of Surgery, University of Michigan School of Medicine, Ann Arbor; 7 Division of Pediatric Surgery, Department of Surgery, University of Cincinnati College of Medicine, Cincinnati, Ohio; 8 Division of Pediatric Surgery, Department of Surgery, University of Wisconsin School of Medicine and Public Health, Madison; 9 Division of Pediatric Surgery, Department of Surgery, Medical College of Wisconsin, Milwaukee; 10 Division of Pediatric Surgery, Department of Surgery, Indiana University School of Medicine, Indianapolis; 11 Division of Pediatric Surgery, Ann & Robert H. Lurie Children’s Hospital of Chicago, Northwestern University Feinberg School of Medicine, Chicago, Illinois; 12 Division of Pediatric Surgery, Department of Surgery, University of Louisville School of Medicine, Louisville, Kentucky

## Abstract

**Importance:**

The factors associated with the failure of nonoperative management of appendicitis and the differences in patient-reported outcomes between successful and unsuccessful nonoperative management remain unknown.

**Objectives:**

To investigate factors associated with the failure of nonoperative management of appendicitis and compare patient-reported outcomes between patients whose treatment succeeded and those whose treatment failed.

**Design, Setting, and Participants:**

This study was a planned subgroup secondary analysis conducted in 10 children’s hospitals that included 370 children aged 7 to 17 years with uncomplicated appendicitis enrolled in a prospective, nonrandomized clinical trial between May 1, 2015, and October 31, 2018, with 1-year follow-up comparing nonoperative management with antibiotics vs surgery for uncomplicated appendicitis. Statistical analysis was performed from November 1, 2019, to February 12, 2022.

**Interventions:**

Nonoperative management with antibiotics vs surgery.

**Main Outcomes and Measures:**

Failure of nonoperative management and patient-reported outcomes. The relative risk (RR) of failure based on sociodemographic and clinical characteristics was calculated. Patient-reported outcomes were compared based on the success or failure of nonoperative management.

**Results:**

Of 370 patients (34.6% of 1068 total patients; 229 boys [61.9%]; median age, 12.3 years [IQR, 10.0-14.6 years]) enrolled in the nonoperative group, treatment failure occurred for 125 patients (33.8%) at 1 year, with 53 patients (14.3%) undergoing appendectomy during initial hospitalization and 72 patients (19.5%) experiencing delayed treatment failure after hospital discharge. Higher patient-reported pain at presentation was associated with increased risk of in-hospital treatment failure (RR, 2.1 [95% CI, 1.0-4.4]) but not delayed treatment failure (RR, 1.3 [95% CI, 0.7-2.3]) or overall treatment failure at 1 year (RR, 1.5 [95% CI, 1.0-2.2]). Pain duration greater than 24 hours was associated with decreased risk of delayed treatment failure (RR, 0.3 [95% CI, 0.1-1.0]) but not in-hospital treatment failure (RR, 1.2 [95% CI, 0.5-2.7]) or treatment failure at 1 year (RR, 0.7 [95% CI, 0.4-1.2]). There was no increased risk of treatment failure associated with age, white blood cell count, sex, race, ethnicity, primary language, insurance status, transfer status, symptoms at presentation, or imaging results. Health care satisfaction at 30 days and patient-reported, health-related quality of life at 30 days and 1 year were not different. Satisfaction with the decision was higher with successful nonoperative management at 30 days (28.0 vs 27.0; difference, 1.0 [95% CI, 0.01-2.0]) and 1 year (28.1 vs 27.0; difference, 1.1 [95% CI, 0.2-2.0]).

**Conclusions and Relevance:**

This analysis suggests that a higher pain level at presentation was associated with a higher risk of initial failure of nonoperative management and that a longer duration of pain was associated with lower risk of delayed treatment failure. Although satisfaction was high in both groups, satisfaction with the treatment decision was higher among patients with successful nonoperative management at 1 year.

**Trial Registration:**

ClinicalTrials.gov Identifier: NCT02271932

## Key Points

**Question:**

What factors are associated with the failure of nonoperative management of appendicitis, and do patient-reported outcomes differ between those whose treatment succeeded vs those whose treatment failed?

**Findings:**

In this planned subgroup analysis of a nonrandomized clinical trial of 1068 patients, higher pain scores at presentation were associated with increased risk of in-hospital treatment failure, and longer pain duration was associated with decreased risk of delayed treatment failure. Although satisfaction was high overall, patients with successful nonoperative management had higher satisfaction with their decision at 1 year than those whose treatment failed.

**Meaning:**

This secondary analysis found that satisfaction with treatment decision was high and that pain level and duration were associated with failure of nonoperative management of appendicitis.

## Teaser

This planned secondary analysis of a nonrandomized clinical trial investigates the factors associated with the failure of nonoperative management of pediatric appendicitis and compares patient-reported outcomes between patients whose treatment succeeded and those whose treatment failed.

## Introduction

Between 60 000 and 80 000 children undergo surgery for the treatment of appendicitis each year, making it the most common indication for emergency intra-abdominal surgery in the pediatric patient population.^1,2^ Despite an increase in the use of laparoscopy for surgical treatment of appendicitis, between 5% and 15% of patients undergoing an appendectomy for uncomplicated appendicitis will experience at least 1 complication, with serious complications occurring for 1% to 7% of patients.^3,4,5,6^ Evidence from several large trials of adults, both in the United States and Europe, has shown that treatment with antibiotics alone is a reasonable alternative to an appendectomy for select patients.^7,8,9,10^ A recent multi-institutional interventional study that included more than 1000 pediatric patients demonstrated that nonoperative management with antibiotics alone is an effective strategy for treating children with uncomplicated acute appendicitis, with a 1-year success rate of 67%, no increase in complications, and fewer days lost to disability compared with surgery.^11^

Accurate risk stratification is an essential component of collaborative decision-making and requires an understanding of patient-specific factors that are associated with the probability of treatment failure. Studies have shown that children who present with a diagnosis of complicated appendicitis or those with an appendicolith are at an increased risk of nonoperative treatment failure, but there is a lack of research exploring the association between patient-specific characteristics at the time of presentation and the risk of unsuccesful nonoperative management for children with uncomplicated appendicitis.^7,12,13^

In addition to medical outcomes, assessment of patient-reported outcomes, including quality of life, health care satisfaction, and satisfaction with treatment decisions, are important in determining the effectiveness of treatment options.^14^ The aim of this study was to investigate the factors associated with the failure of nonoperative management of uncomplicated appendicitis and to compare patient-reported outcomes between patients whose treatment succeeded and those whose treatment failed.

## Methods

### Study Design

This study was a planned subgroup secondary analysis of pediatric patients enrolled in a prospective, nonrandomized, clinical multi-institutional trial between May 1, 2015, and October 31, 2018, with 1-year follow-up investigating nonoperative management for children with uncomplicated appendicitis across 10 children’s hospitals participating in the Midwest Pediatric Surgery Consortium.^11,15^ This study investigates the factors associated with the failure of nonoperative management of appendicitis and compares patient-reported outcomes between patients whose nonoperative management succeeded and those whose nonoperative management failed. The primary results from that study have been published.^11,16^ Institutional review board approval was obtained at each participating site (Nationwide Children’s Hospital, St Louis Children’s Hospital/Washington University School of Medicine in St Louis, University of Chicago Medicine Comer Children’s Hospital, C. S. Mott Children’s Hospital/University of Michigan, Cincinnati Children’s Hospital Medical Center, American Family Children’s Hospital/University of Wisconsin Madison, Children’s Hospital of Wisconsin, Riley Children’s Hospital/Indiana University School of Medicine, Ann & Robert H. Lurie Children’s Hospital of Chicago, and Norton Children’s Hospital/University of Louisville School of Medicine); all caregivers provided written consent, and all children aged 9 years or older provided written assent. The study followed the Transparent Reporting of Evaluations With Nonrandomized Designs (TREND) reporting guideline for nonrandomized clinical trials (trial prototcol in Supplement 1).^17^

Children aged 7 through 17 years with a diagnosis of uncomplicated appendicitis who met the following inclusion criteria were enrolled: (1) imaging-confirmed appendicitis showing an appendix diameter of 1.1 cm or less, no abscess, no appendicolith, and no phlegmon; (2) a white blood cell count between 5000 cells/μL and 18 000 cells/μL (to convert to cells ×10^9^/L, multiply by 0.001); and (3) onset of abdominal pain less than 48 hours prior to initiation of antibiotic therapy. Caregivers of the patients chose either surgery or nonoperative management with antibiotics. Patients were considered to have successfully completed nonoperative treatment if they were initially treated with antibiotics alone and had not undergone an appendectomy by the end of the 1-year follow-up period (Figure).

**Figure.**
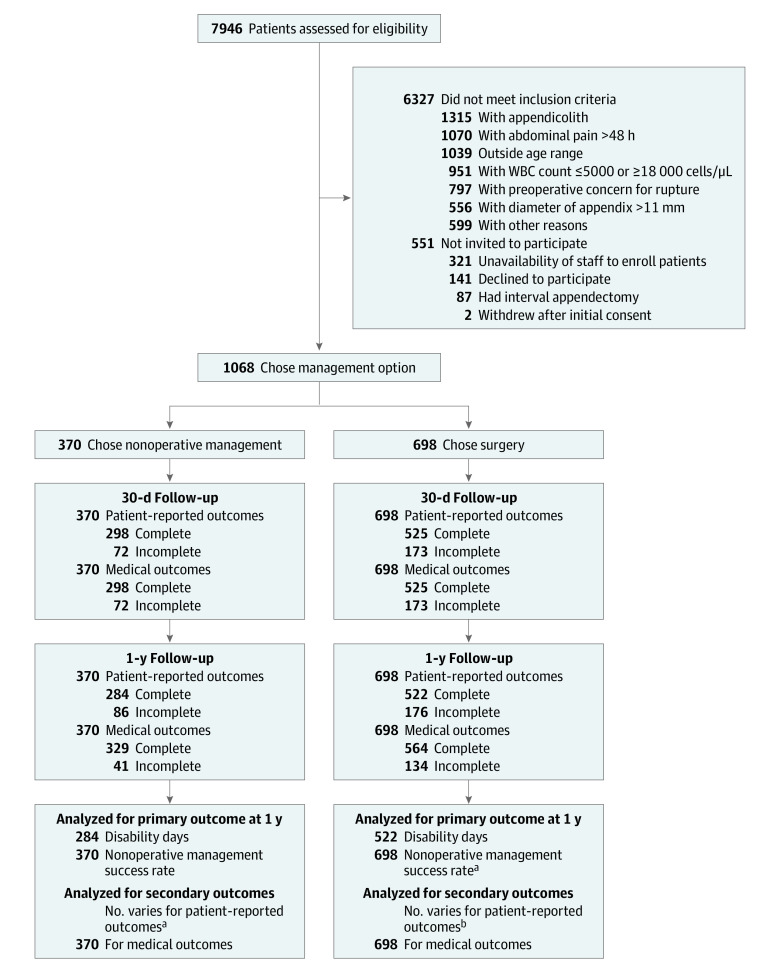
Study Flow of Initial Prospective, Nonrandomized, Clinical Multi-institutional Study Investigating Nonoperative Management and Surgery for Uncomplicated Appendicitis WBC indicates white blood cell. ^a^Although the nonoperative management success rate can be assessed only in the nonoperative management group, the data from all 698 patients in the surgery group were used to perform the inverse probability of treatment weighting analysis for the nonoperative management success rate. ^b^Sample sizes for secondary patient-reported outcomes vary based on availability of completed surveys for each measure. The sample size for each secondary patient-reported outcome is specified throughout the article and in Tables 1 to 4.

Statistical analysis was performed from November 1, 2019, to February 12, 2022. The medical records of all patients treated as part of the nonoperative group were reviewed. Patients’ sociodemographic data were extracted along with data pertaining to clinical characteristics at the time of initial evaluation and the success of nonoperative treatment. Success of nonoperative treatment was defined as not having undergone an appendectomy by 1 year of follow-up. This outcome was evaluated for all children based on information collected from follow-up visits and surveys, medical record review, and primary care clinician follow-up calls. The relative risk (RR) of failure (and the associated 95% CIs) of nonoperative management during the initial admission (in-hospital treatment failure), after hospital discharge through 1 year (delayed treatment failure), and overall treatment failure by 1 year based on patient sociodemographic and clinical characteristics were estimated via modified Poisson regression.^18^ The linear trend of ordinal characteristics in the risk for treatment failure was also tested through these regression models. In-hospital treatment failure was defined as having undergone an appendectomy during the initial hospitalization and included patients who did not improve clinically, patients who worsened clinically, and patients who were transferred to the operative group based on caregiver choice.

Patient-reported outcomes, including health-related quality of life at 30 days and at 1 year (scale, 0-100, where higher scores indicate greater quality of life), health care satisfaction at 30 days (scale, 0-100, where higher scores indicate greater satisfaction), and satisfaction with the initial treatment decision at 30 days and at 1 year (scale, 0-30, where higher scores indicate greater satisfaction), were compared based on the success or failure of nonoperative management at 1 year through 2-sample *t* tests and the differences in mean values and 95% CIs, assuming that the unequal variance of group mean values was calculated. Patient-reported outcomes could be evaluated only for patients or caregivers who completed the follow-up surveys at the specified time point. The size of the cohort available for each comparison is denoted in Table 1, Table 2, Table 3, and Table 4. Data management and analyses were conducted in Stata, version 15.0 (StataCorp LLC).

**Table 1.** Associations Between Patient Demographic and Clinical Characteristics With In-Hospital Failure of Nonoperative Management of Appendicitis

Characteristic	Patients, No. (%)		Relative risk of failure (95% CI)
	Successful hospital discharge (n = 317)	In-hospital treatment failure (n = 53)	
Age, y^a^			
Median (IQR)	12.3 (10.0-14.6)	12.8 (10.3-14.7)	NA
≤10	117 (88.0)	16 (12.0)	1 [Reference]
11-14	133 (83.1)	27 (16.9)	1.4 (0.8-2.5)
15-17	67 (87.0)	10 (13.0)	1.1 (0.5-2.3)
Pain score^a^^,^^b^			
Median (IQR)	5 (3-7)	6 (4-8)	NA
0-2	67 (89.3)	8 (10.7)	1 [Reference]
3-6	135 (87.7)	19 (12.3)	1.2 (0.5-2.5)
7-10	83 (77.6)	24 (22.4)	2.1 (1.0-4.4)^c^
Missing	32	2	NA
Duration of pain at ED presentation, h^a^			
Median (IQR)	16 (11-24)	20 (10-24)	NA
<10	64 (86.5)	10 (13.5)	1 [Reference]
10-18	109 (88.6)	14 (11.4)	0.8 (0.4-1.8)
19-24	93 (83.0)	19 (17.0)	1.3 (0.6-2.6)
25-72	47 (83.9)	9 (16.1)	1.2 (0.5-2.7)
Missing	4	1	NA
White blood cell count, ×1000 cells^a^			
Median (IQR)	12.5 (10.4-14.9)	11.9 (10.2-15.7)	NA
≤12.0	143 (84.1)	27 (15.9)	1 [Reference]
12.1-15.0	102 (90.3)	11 (9.7)	0.6 (0.3-1.2)
15.1-24.0	71 (82.6)	15 (17.4)	1.1 (0.6-2.0)
Missing	0	1	NA
Sex			
Boys	196 (85.6)	33 (14.4)	1 [Reference]
Girls	121 (85.8)	20 (14.2)	1.0 (0.6-1.7)
Race			
Black	29 (82.9)	6 (17.1)	1.2 (0.5-2.6)
White	236 (85.5)	40 (14.5)	1 [Reference]
Other^d^	46 (86.8)	7 (13.2)	0.9 (0.4-1.9)
Not reported or not documented	6	0	NA
Ethnicity			
Hispanic or Latino	32 (86.5)	5 (13.5)	1.0 (0.4-2.5)
Not Hispanic or Latino	211 (86.8)	32 (13.2)	1 [Reference]
Other^e^	71 (82.6)	15 (17.4)	1.3 (0.8-2.3)
Not documented or not reported	3	1	NA
Primary language			
English	244 (85.9)	40 (14.1)	1 [Reference]
Other language	59 (85.5)	10 (14.5)	1.0 (0.5-2.0)
Not documented or not reported	14	3	NA
Insurance			
Private insurance	217 (87.2)	32 (12.9)	0.7 (0.4-1.2)
All other insurance or no insurance	98 (82.4)	21 (17.7)	1 [Reference]
Medicaid	90 (82.6)	19 (17.4)	NA
Other or no insurance	8 (80.0)	2 (20.0)	NA
Not documented or not reported	2	0	NA
Transfer from another ED or hospital^f^	139 (87.4)	20 (12.6)	0.8 (0.5-1.4)
Symptoms at presentation^f^			
Nausea	196 (86.0)	32 (14.0)	0.9 (0.6-1.6)
Emesis	134 (84.8)	24 (15.2)	1.1 (0.7-1.8)
Diarrhea	35 (89.7)	4 (10.3)	0.7 (0.3-1.8)
Fever	59 (83.1)	12 (16.9)	1.2 (0.7-2.2)
Anorexia	144 (85.7)	24 (14.3)	1.0 (0.6-1.6)
Imaging performed			
Ultrasongraphy only	224 (83.9)	43 (16.1)	2.4 (1.0-5.8)
CT scan only	69 (93.2)	5 (6.8)	1 [Reference]
Ultrasonography and CT scan	23 (82.1)	5 (17.9)	2.6 (0.8-8.5)
Missing	1	0	NA

Abbreviations: CT, computed tomography; ED, emergency department; NA, not applicable.

^a^ The *P* value for linear trend in risk over categorical groups is as follows: age, *P* = .67; pain score, *P* = .03; pain duration, *P* = .39; and white blood cell count, *P* = .99.

^b^ Scale from 1 to 10; higher scores indicates more pain.

^c^ Significant association at *P* < .05.

^d^ Other includes Asian, American Indian, Alaska Native, other race, and biracial or multiracial.

^e^ The Other category included caregiver-entered free-text responses of American, Albanian, Asian, Middle Eastern, Nepali, Somali, and multiethnic.

^f^ Relative risk association with not having characteristic.

**Table 2.** Associations Between Patient Demographic and Clinical Characteristics With Development of Recurrent Appendicitis Between Discharge and 1 Year

Characteristic	Patients, No. (%)		Relative risk of recurrence (95% CI)
	Continued treatment success to 1 y (n = 245)	Recurrence between hospital discharge and 1 y (n = 72)	
Age, y^a^			
Median (IQR)	12.5 (10.4-14.8)	11.1 (9.5-13.7)	NA
≤10	85 (72.7)	32 (27.4)	1 [Reference]
11-14	104 (78.2)	29 (21.8)	0.8 (0.5-1.2)
15-17	56 (83.6)	11 (16.4)	0.6 (0.3-1.1)
Pain score^a^^,^^b^			
Median (IQR)	5 (3-7)	5 (3-8)	NA
0-2	53 (79.1)	14 (20.9)	1 [Reference]
3-6	106 (78.5)	29 (21.5)	1.0 (0.6-1.8)
7-10	61 (73.5)	22 (26.5.)	1.3 (0.7-2.3)
Missing	25	7	NA
Duration of pain at ED presentation, h^a^			
Median (IQR)	17 (12-24)	12 (10-24)	NA
<10	48 (75.0)	16 (25.0)	1 [Reference]
10-18	79 (72.5)	30 (27.5)	1.1 (0.7-1.9)
19-24	73 (78.5)	20 (21.5)	0.9 (0.5-1.5)
25-72	43 (91.5)	4 (8.5)	0.3 (0.1-1.0)^c^
Missing	2	2	NA
White blood cell count, ×1000 cells^a^			
Median (IQR)	12.5 (10.3-14.9)	12.7 (10.4-14.8)	NA
≤12.0	114 (79.7)	29 (20.3)	1 [Reference]
12.1-15.0	74 (72.6)	28 (27.5)	1.4 (0.9-2.1)
15.1-24.0	57 (80.3)	14 (19.7)	1.0 (0.6-1.7)
Missing	0	1	NA
Sex			
Boys	155 (79.1)	41 (20.9)	1 [Reference]
Girls	90 (74.4)	31 (25.6)	1.2 (0.8-1.8)
Race			
Black	20 (69.0)	9 (31.0)	1.5 (0.8-2.7)
White	186 (78.8)	50 (21.2)	1 [Reference]
Other^d^	33 (71.7)	13 (28.3)	1.3 (0.8-2.3)
Not reported or not documented	6	0	NA
Ethnicity			
Hispanic or Latino	23 (71.9)	9 (28.1)	1.3 (0.7-2.5)
Not Hispanic or Latino	167 (79.2)	44 (20.9)	1 [Reference]
Other^e^	52 (73.2)	19 (26.8)	1.3 (0.8-2.1)
Not documented or not reported	3	0	NA
Primary language			
English	189 (77.5)	55 (22.5)	1 [Reference]
Other language	45 (76.3)	14 (23.7)	1.1 (0.6-1.8)
Not documented or not reported	11	3	NA
Insurance			
Private insurance	173 (79.7)	44 (20.3)	0.7 (0.5-1.1)
All other insurance or no insurance	71 (72.5)	27 (27.6)	1 [Reference]
Medicaid	66	24	NA
Other or no insurance	5	3	NA
Not documented or not reported	1	1	NA
Transfer from another ED or hospital^f^	105 (75.5)	34 (24.5)	1.1 (0.8-1.7)
Symptoms at presentation^f^			
Nausea	149 (76.0)	47 (24.0)	1.2 (0.8-1.8)
Emesis	100 (74.6)	34 (25.4)	1.2 (0.8-1.8)
Diarrhea	30 (85.7)	5 (14.3)	0.6 (0.3-1.4)
Fever	49 (83.1)	10 (17.0)	0.7 (0.4-1.3)
Anorexia	109 (75.7)	35 (24.3)	1.1 (0.8-1.7)
Imaging performed			
Ultrasonography only	173 (77.2)	51 (22.8)	0.9 (0.6-1.4)
CT scan only	51 (73.9)	18 (26.1)	1 [Reference]
Ultrasonography and CT scan	20 (87.0)	3 (13.0)	0.5 (0.2-1.6)
Missing	1	0	NA

Abbreviations: CT, computed tomography; ED, emergency department; NA, not applicable.

^a^ The *P* value for linear trend in risk over categorical groups is as follows: age, *P* = .09; pain score, *P* = .40; pain duration, *P* = .02; and white blood cell count, *P* = .84.

^b^ Scale from 1 to 10; higher scores indicates more pain.

^c^ Significant association at *P* < .05.

^d^ Other includes Asian, American Indian, Alaska Native, other race, and biracial or multiracial.

^e^ The Other category included caregiver-entered free-text responses of American, Albanian, Asian, Middle Eastern, Nepali, Somali, and multiethnic.

^f^ Relative risk association with not having characteristic.

**Table 3.** Associations Between Patient Demographic and Clinical Characteristics With Overall Treatment Failure of Nonoperative Management at 1 Year

Characteristic	Patients, No. (%)		Relative risk of failure at 1 y (95% CI)
	Treatment success at 1 y (n = 245)	Treatment failure at 1 y (n = 125)	
Age, y^a^			
Median (IQR)	12.5 (10.4-14.8)	12.0 (9.7-14.3)	NA
≤10	85 (63.9)	48 (36.1)	1 [Reference]
11-14	104 (65.0)	56 (35.0)	1.0 (0.7-1.3)
15-17	56 (72.7)	21 (27.3)	0.8 (0.5-1.2)
Pain score^a^^,^^b^			
Median (IQR)	5 (3-7)	6 (3.5-8)	NA
0-2	53 (70.7)	22 (29.3)	1 [Reference]
3-6	106 (68.8)	48 (31.2)	1.1 (0.7-1.6)
7-10	61 (57.0)	46 (43.0)	1.5 (1.0-2.2)
Missing	25	9	NA
Duration of pain at ED presentation, h^a^			
Median (IQR)	17 (12-24)	13 (10-24)	NA
<10	48 (64.9)	26 (35.1)	1 [Reference]
10-18	79 (64.2)	44 (35.8)	1.0 (0.7-1.5)
19-24	73 (65.2)	39 (34.8)	1.0 (0.7-1.5)
25-72	43 (76.8)	13 (23.2)	0.7 (0.4-1.2)
Missing	2	3	NA
White blood cell count, ×1000 cells^a^			
Median (IQR)	12.5 (10.3-14.9)	12.6 (10.3-14.8)	NA
≤12.0	114 (67.1)	56 (32.9)	1 [Reference]
12.1-15.0	74 (65.5)	39 (34.5)	1.0 (0.8-1.5)
15.1-24.0	57 (66.3)	29 (33.7)	1.0 (0.7-1.5)
Missing	0	1	NA
Sex			
Boys	155 (67.7)	74 (32.3)	1 [Reference]
Girls	90 (63.8)	51 (36.2)	1.1 (0.8-1.5)
Race			
Black	20 (57.1)	15 (42.9)	1.3 (0.9-2.0)
White	186 (67.4)	90 (32.6)	1 [Reference]
Other^c^	33 (62.3)	20 (37.7)	1.2 (0.8-1.7)
Not reported or not documented	6	0	NA
Ethnicity			
Hispanic or Latino	23 (62.2)	14 (37.8)	1.2 (0.8-1.9)
Not Hispanic or Latino	167 (68.7)	76 (31.3)	1 [Reference]
Other^d^	52 (60.5)	34 (39.5)	1.3 (0.9-1.7)
Not documented or not reported	3	1	NA
Primary language			
English	189 (66.6)	95 (33.5)	1 [Reference]
Other language	45 (65.2)	24 (34.8)	1.0 (0.7-1.5)
Not documented or not reported	11	6	NA
Insurance			
Private insurance	173 (69.5)	76 (30.5)	0.8 (0.6-1.0)
All other insurance or no insurance	71 (59.7)	48 (40.3)	1 [Reference]
Medicaid	66	43	NA
Other or no insurance	5	5	NA
Not documented or not reported	1	1	NA
Transfer from another ED or hospital^e^	105 (66.0)	54 (34.0)	1.0 (0.8-1.3)
Symptoms at presentation^e^			
Nausea	149 (65.4)	79 (34.7)	1.1 (0.8-1.4)
Emesis	100 (63.3)	58 (36.7)	1.2 (0.9-1.5)
Diarrhea	30 (76.9)	9 (23.1)	0.7 (0.4-1.2)
Fever	49 (69.0)	22 (31.0)	0.9 (0.6-1.3)
Anorexia	109 (64.9)	59 (35.1)	1.1 (0.8-1.4)
Imaging performed			
Ultrasonography only	173 (64.8)	94 (35.2)	0.9 (0.8-1.1)
CT scan only	51 (68.9)	23 (31.1)	1 [Reference]
Ultrasonography and CT scan	20 (71.4)	8 (28.6)	1.0 (0.8-1.4)
Missing	1	0	NA

Abbreviations: CT, computed tomography; ED, emergency department; NA, not applicable.

^a^ The *P* value for linear trend in risk over categorical groups is as follows: age, *P* = .22; pain score, *P* = .04; pain duration, *P* = .19; and white blood cell count, *P* = .87.

^b^ Scale from 1 to 10; higher scores indicates more pain.

^c^ Other includes Asian, American Indian, Alaska Native, other race, and biracial or multiracial.

^d^ The Other category included caregiver-entered free-text responses of American, Albanian, Asian, Middle Eastern, Nepali, Somali, and multiethnic.

^e^ Relative risk association with not having characteristic.

**Table 4.** Patient-Reported Outcomes Based on Overall Success or Failure of Nonoperative Management at 1 Year

Outcome	Mean (SD)		Difference (95% CI)
	Treatment success at 1 y (n = 245)	Treatment failure at 1 y (n = 125)	
PedsQL health care satisfaction score (30 d)^a^	93.2 (13.0)	91.9 (13.7)	1.3 (−2.0 to 4.6)
Satisfaction with decision score (30 d)^b^	28.0 (3.8)	27.0 (4.2)	1.0 (0.01 to 2.0)^c^
Satisfaction with decision score (1 y)^d^	28.1 (3.8)	27.0 (3.7)	1.1 (0.2 to 2.0)^c^
PedsQL child QOL (30 d)^e^	89.4 (10.3)	87.9 (10.6)	1.5 (−1.2 to 4.1)
PedsQL parent proxy for child QOL (30 d)^f^	90.2 (9.8)	86.9 (11.8)	3.3 (0.5 to 6.0)^c^
PedsQL child QOL (1 y)^g^	90.5 (9.9)	90.6 (8.6)	−0.1 (−2.4 to 2.2)
PedsQL parent proxy for child QOL (1 y)^h^	90.9 (9.6)	91.9 (8.5)	−1.0 (−3.2 to 1.2)

Abbreviations: PedsQL, Pediatric Quality of Life Inventory; QOL, quality of life.

^a^ Available for 295 (198 with treatment success and 97 with treatment failure).

^b^ Available for 296 (198 with treatment success and 98 with treatment failure).

^c^ Significant association at *P* < .05.

^d^ Available for 280 (183 with treatment success and 97 with treatment failure).

^e^ Available for 274 (184 with treatment success and 90 with treatment failure).

^f^ Available for 271 (179 with treatment success and 92 with treatment failure).

^g^ Available for 292 (196 with treatment success and 96 with treatment failure).

^h^ Available for 280 (183 with treatment success and 97 with treatment failure).

## Results

### Enrollment

Of the 1068 patients enrolled, the 370 (34.6%; 229 boys [61.9%]; median age, 12.3 years [IQR, 10.0-14.6 years]) who chose nonoperative management are included in this subgroup analysis. Complete follow-up was available for 329 of 370 patients (88.9%). Additional demographic information for the cohort has been previously published.^11^ Of the 370 patients who chose an initial nonoperative management strategy, 53 (14.3%) experienced early treatment failure and underwent an appendectomy during the initial hospital admission. The most common reason for undergoing appendectomy was treatment failure (25 of 53 [47.2%]), followed by clinical worsening (17 of 53 [32.1%], 4 of whom also experienced treatment failure), caregiver decision (16 of 53 [30.2%]; 1 also had clinical worsening, and 5 experienced treatment failure), and not meeting hospital discharge criteria within 48 hours (6 of 53 [11.3%]; 2 also had clinical worsening, and 1 experienced treatment failure); 2 patients had other unspecified reasons. In addition to the 53 patients who underwent appendectomy during the initial hospitalization, an additional 72 patients (19.5%) experienced delayed treatment failure and underwent an appendectomy within 1 year of treatment, representing an overall 1-year failure rate of 33.8% (125 of 370). The median time to recurrence for those who were discharged from the hospital was 2 months (IQR, 0-5 months).

### Associations of Sociodemographic and Clinical Characteristics With Treatment Failure

During the initial hospital admission, a pain score between 7 and 10 at the time of admission was associated with a more than doubled risk of in-hospital treatment failure compared with a pain score between 0 and 2 (RR, 2.1 [95% CI, 1.0-4.4]; *P* = .03 for linear trend) (Table 1). Higher patient-reported pain at presentation was not associated with delayed treatment failure (RR, 1.3 [95% CI, 0.7-2.3]; Table 2) or overall treatment failure at 1 year (RR, 1.5 [95% CI, 0.97-2.22]; Table 3). The patient’s age, sex, race, ethnicity, insurance status, or primary language spoken were not associated with an increased risk of in-hospital treatment failure (Table 1). The duration of reported pain at the time of presentation to the emergency department, the patient’s white blood cell count, transfer status, and symptoms at presentation were also not associated with an increased risk of early treatment failure. Although not statistically significant, patients who received imaging only by ultrasonography were more than twice as likely to experience in-hospital treatment failure (RR, 2.4 [95% CI, 1.0-5.8]) compared with those who received only a computed tomography scan (Table 1).

Duration of pain longer than 24 hours was associated with a decreased risk of delayed treatment failure (RR, 0.3 [95 CI, 0.1-1.0]; *P* = .02 for linear trend) (Table 2) but not in-hospital treatment failure (RR, 1.2 [95% CI, 0.5-2.7]) (Table 1) or treatment failure at 1 year (RR, 0.7 [95 CI, 0.4-1.2]; *P* = .19 for linear trend) (Table 3). Increasing pain score category during the initial hospitalization was associated with a small increased risk of delayed treatment failure. No other sociodemographic or clinical characteristics at the time of presentation, including age, white blood cell count, pain score, sex, race, ethnicity, primary language spoken in the home, insurance status, transfer status, symptoms at presentation, or imaging performed, were associated with either delayed treatment failure after hospital discharge (Table 2) or overall treatment failure at 1 year (Table 3).

### Patient-Reported Outcomes Based on Success of Nonoperative Management

Reported quality-of-life score, health care satisfaction, and caregiver satisfaction with the treatment decision were high across both groups (Table 4). The mean (SD) health care satisfaction scores at 30 days (93.2 [13.0] vs 91.9 [13.7]) and the mean (SD) health-related quality of life scores reported by the children at 30 days (89.4 [10.3] vs 87.9 [10.6]) and 1 year (90.5 [9.9] vs 90.6 [8.6]) were not different between patients with successful and patients with unsuccessful nonoperative management at 1 year. Although satisfaction was high overall, the mean (SD) satisfaction with decision scores were higher for patients with successful nonoperative management at 30 days (28.0 [3.8] vs 27.0 [4.2]; difference, 1.0 [95% CI, 0.01-2.0]) and 1 year (28.1 [3.8] vs 27.0 [3.7]; difference, 1.1 [95% CI, 0.2-2.0]).

## Discussion

Among children undergoing nonoperative management of uncomplicated appendicitis, higher initial pain scores at presentation were associated with an increased risk of in-hospital treatment failure but not with development of delayed treatment failure after hospital discharge or overall treatment failure at 1 year. Duration of pain longer than 24 hours at presentation was associated with decreased risk of delayed treatment failure but not in-hospital treatment failure or treatment failure at 1 year. Other sociodemographic and clinical characteristics, including age, sex, race, ethnicity, primary language spoken in the home, insurance status, white blood cell count, transfer status, imaging, or symptoms at presentation, were not associated with an increased risk of treatment failure. Health care satisfaction, health-related quality of life, and satisfaction with decision scores were high for all patients, but patients with successful nonoperative management at 1 year had higher satisfaction with decision scores than those whose nonoperative management failed.

Clinicians can increase the perceived value of care for children presenting with uncomplicated acute appendicitis by engaging families in a collaborative discussion about their treatment options, including the possibility of pursuing a nonoperative treatment strategy. Initial treatment with antibiotics alone is successful for nearly 70% of patients, carries similar risks of complication as appendectomy, and is associated with fewer disability days at 1 year.^11^ In our study, the families that elected nonoperative management reported high quality-of-life scores and remained satisfied with their health care decision at both 30 days and 1 year. Optimal engagement in health care decision-making relies on providing accurate expectations to patients and families regarding the probability of treatment success as well as discussing options for treatment failure.

It is important to continue evaluating the association between patient-specific factors and treatment failure to better refine the criteria for identifying patients most likely to achieve a successful outcome from nonoperative management. Patients with complicated appendicitis and those with an appendicolith are at an increased risk of treatment failure with antibiotics alone; therefore, these patients should be excluded from nonoperative treatment.^12,13^ For all other pediatric patients with uncomplicated acute appendicitis, nonoperative treatment should be viewed as an equitable alternative to surgery.^11,16^ The risk of treatment failure at 1 year for patients in our study electing to pursue nonoperative treatment for uncomplicated appendicitis was not associated with a patient’s age, race, ethnicity, insurance status, transfer status, duration of pain at presentation, symptoms at presentation, or white blood cell count. Although the patient-reported intensity of pain at presentation was associated with in-hospital treatment failure, it was not associated with treatment failure at 1 year. It is possible that increased intensity of pain at presentation is associated with a higher degree of disease severity that is less likely to respond to nonoperative management, resulting in early in-hospital treatment failure but not necessarily delayed treatment failure because patients with delayed treatment failure may present with less severe disease that is initially amenable to nonoperative management. This type of association has previously been demonstrated in the orthopedic patient population, where studies have demonstrated that increased preoperative pain was associated with greater disease severity, the decision to pursue surgical management, and the ability to benefit from surgical intervention.^19,20^ Although the present study did not specifically evaluate the association between pain intensity at presentation and treatment choice, our previously published results demonstrated no difference in pain scores at presentation between patients who chose nonoperative management and patients who chose operative management.^11^ All patients who are discharged from the hospital after initiating a nonoperative management strategy should be informed of the risk of recurrence and counseled on the need for surgery should symptoms recur.

Several recent studies have also investigated the factors associated with the failure or success of nonoperative management of uncomplicated acute appendicitis. In a secondary analysis of a large, randomized clinical trial of adults, Haijanen and colleagues^21^ identified an appendiceal diameter of 15 mm or more detected on computed tomography scans and a body temperature higher than 38° C as factors associated with in-hospital failure of nonoperative management. Shay et al^22^ identified a 6.9% in-hospital failure rate and a 20.8% overall failure rate of nonoperative management of adults using a protocol that limited nonoperative management to afebrile patients with less than 3 days of abdominal pain, a white blood cell count less than 15 000 cells/μL, a C-reactive protein level less than 5 mg/dL (to convert to milligrams per liter, multiply by 10.0), an appendiceal diameter less than 1 cm, and no appendicolith detected on imaging. Our study inclusion criteria already incorporated most of these factors associated with treatment failure because we limited our patient population to those with abdominal pain for less than 48 hours, a white blood cell count less than 18 000 cells/μL, an appendiceal diameter of 11 mm or less, and no appendicolith detected on imaging. We did identify an association of duration of pain for more than 24 hours prior to presentation with a decreased risk of delayed treatment failure. A potential explanation for this association is that patients who present later after the onset of pain may have a slower increase in intensity of pain, which may be a marker of more indolent cases of appendicitis that are more amenable to successful nonoperative management.

Although it did not reach statistical significance, the association between imaging solely with ultrasonography and increased risk of in-hospital treatment failure may warrant further investigation. This association may be due to the less detailed nature of ultrasography findings compared with computed tomography scans. More subtle signs of advanced appendicitis are not likely to be detected with nonoperative management and may not be routinely detected or appreciated using ultrasonography. This conclusion is supported by a recent pediatric study that identified the presence of an appendiceal mucosal ulceration on an ultrasonogram as a factor associated with the failure of nonoperative management.^23^ This ultrasographic characteristic was not routinely observed in our study population. Because ultrasonography is the primary imaging modality for children with suspected appendicitis, additional studies exploring the ultrasonographic characteristics associated with the failure of nonoperative management are needed.

Families have an important role in determining the success of treatment, as highlighted by the fact that more than 30% of patients in our study who underwent appendectomy during the initial hospitalization did so because of caregiver choice.^24^ An effective patient-clinician relationship requires trust, which can be facilitated by establishing expectations early in the process. Families need to be informed that nearly 25% of patients who meet initial hospital discharge criteria after initiating nonoperative treatment for acute appendicitis will ultimately undergo an appendectomy within 1 year. If treatment failure does occur, it is likely to be within the first few months because the median time to surgery for patients whose nonoperative management failed after hospital discharge was only 2 months. Providing caregivers with reasonable expectations can help build trust in a collaborative discussion and potentially increase the effectiveness of the treatment by increasing a caregiver’s confidence in the initial decision.

### Limitations

This study has some limitations. The data for this study were collected as part of a multi-institutional patient-choice trial. The decision to allow patients to choose their treatment was made based on evidence from previous studies showing that families are often unwilling to allow their children to be randomly assigned to treatment.^24,25^ Use of a nonrandomized choice design may be more reflective of typical clinical practice and likely led to an increase in enrollment, but it may have also affected the generalizability of the results by introducing treatment selection bias. In addition, complete follow-up was available for 89% of patients who elected nonoperative management, leading to the possibility that loss to follow-up may have affected the results. Last, this study was conducted across 10 tertiary pediatric hospitals in the Midwest; therefore, the patient population may not be reflective of the general US population.

## Conclusions

This study suggests that pediatric patients with uncomplicated acute appendicitis should be offered treatment options, including nonoperative management. Treatment with antibiotics alone is a safe and equitable option for children, with no increased risk of treatment failure based on sociodemographic or objective clinical characteristics at presentation. Families need to be made aware that treatment failure is not uncommon, and they should be provided with anticipatory guidance on how to proceed should symptoms recur. Future research is needed to better define the patient-specific factors associated with the probability of treatment success of nonoperative management of uncomplicated appendicitis.

## Notes

Supplement 1.
Trial Protocol

Supplement 2.
Nonauthor Collaborators

Supplement 3.
Data Sharing Statement
