# Supplementary material for: Demographic and Clinical Characteristics Associated With the Failure of Nonoperative Management of Uncomplicated Appendicitis in Children: Secondary Analysis of a Nonrandomized Clinical Trial
Source: JAMA Netw Open. 2022 May 2;5(5):e229712. doi: 10.1001/jamanetworkopen.2022.9712 (PMC9062687; doi:10.1001/jamanetworkopen.2022.9712)
Supplement: Supplement 1. — Trial Protocol [file jamanetwopen-e229712-s001.pdf]

Title: Multi-Institutional Trial of Non-Operative Management of Uncomplicated Pediatric Appendicitis

Protocol Number: IRB14-00651

Protocol Date: 2/12/2020

Version Number: 13

TABLE OF CONTENTS

ABBREVIATIONS AND DEFINITIONS OF TERMS 4

ABSTRACT 5

1 BACKGROUND INFORMATION AND RATIONALE 6
- 1.1 Introduction 6
- 1.2 Relevant literature and data 6
- 1.3 Compliance Statement 7

2 STUDY OBJECTIVES 8
- 2.1 Primary Objectives 8
- 2.2 Secondary Objectives 8

3 INVESTIGATIONAL PLAN 8
- 3.1 Study Design 8
- 3.2 Study Duration and Enrollment 9
  - 3.2.1 Duration of Study and Enrollment 9
  - 3.2.2 Total Number of Subjects Projected and Site 9
- 3.3 Study Population 9
  - 3.3.1 Inclusion Criteria 10
  - 3.3.2 Exclusion Criteria 10

4 STUDY PROCEDURES 10
- 4.1 Screening and Enrollment 10
- 4.2 Study Procedures: Non-Operative Group 11
  - 4.2.1 Hospital Course 11
  - 4.2.2 Follow-Up 12
- 4.3 Study Procedures: Surgery Group 14
  - 4.3.1 Hospital Course 14
  - 4.3.2 Follow-Up 15

5 STATISTICAL CONSIDERATIONS 16
- 5.1 Primary and Secondary Endpoints 16
- 5.2 Statistical Methods 17
- 5.3 Sample Size and Power 21

6 STUDY ADMINISTRATION 21
- 6.1 Data Collection and Management 22
- 6.2 Confidentiality 23
- 6.3 Regulatory and Ethical Considerations 23
  - 6.3.1 Risk Assessment 23
  - 6.3.2 Potential Benefits of Trial Participation 24
  - 6.3.3 Risk-Benefit Assessment/Risk Minimization 24
  - 6.3.4 Data Safety and Monitoring 25
  - 6.3.5 Adverse events (AE) and Serious adverse events (SAEs) 26
- 6.4 Recruitment Strategy 27

6.5 Informed Consent and Assent ... 27
6.6 Payment to Subjects and Families ... 27
6.7 Confidentiality ... 27

APPENDIX: Nationwide Children's Hospital Standard Nursing Protocol ... 28

APPENDIX: Physician Informed Consent/Assent Script ... 29

APPENDIX: Physician Pros and Cons Script ... 30

APPENDIX: Sample patient handout ... 31

REFERENCES ... 33

ABBREVIATIONS AND DEFINITIONS OF TERMS

CA	Complex appendicitis (ruptured or gangrenous)
CI	Confidence Interval
CIPP	Center for Innovation in Pediatric Practice
CSOR	Center for Surgical Outcomes Research
ED	Emergency Department
NCH	Nationwide Children’s Hospital
OR	Operating Room
PEDSQL	The Pediatric Quality of Life Inventory Measurement Model, a validated pediatric model for measuring quality of life
QOL	Quality of Life
REDCap	The Research Electronic Data Capture System, an electronic system for storing and managing research data in an encrypted form appropriate for clinical information
RINCH	The Research Institute at Nationwide Children’s Hospital
SA	Simple appendicitis

ABSTRACT

Traditionally, children presenting with appendicitis are referred for urgent appendectomy. Recent improvements in both the quality and availability of diagnostic imaging now allow for better pre-operative characterization of appendicitis, including the severity of inflammation; size of the appendix; and presence of extra-luminal inflammation, phlegmon, or abscess [1-3]. These imaging advances, in conjunction with the availability of broad spectrum oral antibiotics, allow for the identification of a subset of patients with uncomplicated appendicitis that can be successfully treated with antibiotics alone.

Several recent European randomized controlled trials demonstrated that therapy with antibiotics alone is an effective treatment option for adults with appendicitis, with no increase in the rate of complicated appendicitis. A study from Nationwide Children's Hospital (NCH) in Columbus, Ohio, demonstrated the effectiveness of a non-operative treatment strategy in children with suspected uncomplicated appendicitis [4]. At interim analysis with a median follow-up of nine months, non-operative management had a 78% success rate with no increase in the rate of complicated appendicitis (non-operative management group 3% vs. surgery group 11%).

The objective of this study is to perform a multi-institutional trial across the ten hospitals of the Midwest Pediatric Surgical Research Consortium to investigate the effectiveness of non-operative management for uncomplicated acute appendicitis as an alternative first line therapy in children across a group of large children's hospitals. Patients diagnosed with uncomplicated appendicitis without a fecalith at participating institutions between September 2014 and October 2018 will be offered a choice of non-operative management or appendectomy. Outcomes will include determining the success rate of non-operative management and comparing differences in disability days, complication rates, cost of care, and quality of life between patients choosing non-operative management and those choosing appendectomy.

Patients diagnosed with uncomplicated appendicitis without a fecalith between September 2014 and October2018 will choose between non-operative management and appendectomy. Data to be collected include patient demographics, clinical information related to the diagnosis and hospital admission, and patient-centered quality of life measures. In addition, we will collect detailed cost information related to the treatment and care for appendicitis and post-surgery care, reported by participating institutions and self-reported by patients. For those subjects who have consented, a one year follow-up phone call or letter (with survey) with the patient's PCP will be performed . Long-term follow-up will be conducted by phone or email to collect information on post-treatment morbidity, including disability days, and healthcare satisfaction. All data will be collected in a central Research Electronic Data Capture (REDCap) database housed at NCH.

1 BACKGROUND INFORMATION AND RATIONALE

1.1 Introduction

Over 70,000 appendectomies are performed on children annually in the United States for the diagnosis of appendicitis. Irrespective of the severity of the inflammation and the presence or absence of extraluminal extension of disease, appendicitis has been traditionally treated with an operation, hospital admission, and a variable course of antibiotics. However, operative management exposes children to the risks of anesthesia and surgery, and is associated with inherent post-operative pain and stress.

Several recent European randomized controlled trials demonstrated that therapy with antibiotics alone is an effective treatment option for adults with appendicitis [1-3, 5-7] with no increase in the rate of complicated appendicitis [8]. An ongoing prospective clinical trial from NCH confirmed the effectiveness of a non-operative treatment strategy in children with suspected uncomplicated appendicitis [4]. At 1 year, non-operative management has a 76% success rate with no increase in the rate of complicated appendicitis (non-operative management group 3% vs. surgery group 11%).

Our objective is to validate these findings at multiple institutions to determine the success rate of non-operative management and compare differences in disability days, complication rates, cost of care, and quality of life between patients choosing non-operative management and those choosing appendectomy.

1.2 Relevant literature and data

The management of appendicitis has evolved to incorporate alternative treatment strategies based on severity of illness, in part because of substantial improvements in imaging modalities. In patients with perforated appendicitis, treatment algorithms now utilize radiographic catheter-guided procedures for intra-abdominal abscesses, and non-operative management with prolonged antibiotics for patients with intra-abdominal phlegmons and prolonged symptoms for whom a surgical resection may incur greater peri-operative morbidity [9-11]. Currently, investigators are also re-evaluating the need to perform an interval appendectomy after the child recovers from their acute illness in these severe cases. [12-15]

To date, uncomplicated appendicitis in the United States is treated with appendectomy, brief peri-operative antibiotics, and a short hospital stay for post-operative observation. Operative management is curative in these cases, but overall complication rates are reported between 5-15% and with serious complications occurring in 1.5-2% of patients [16-19]. In addition, operative management exposes children to the risks of anesthesia and is associated with inherent post-operative pain [20] and stress. Furthermore, the median total cost of an uncomplicated appendectomy in children is around $6,355 [21]. With the rate of negative appendectomies at approximately 6.7% [22] this indicates that over $29 million of the above costs are unnecessary. A successful non-operative treatment strategy for the management of uncomplicated appendicitis would decrease the number of children requiring appendectomy.

There have been several randomized controlled studies comparing appendectomy to antibiotics in patients with appendicitis (in non-U.S. countries) [1-3, 5-7]. A meta-analysis of 4 of these studies reveals that antibiotic use is a safe initial treatment for appendicitis and that non-

operative management of appendicitis is associated with a significantly lower risk of complications with no difference in risk of developing complicated appendicitis [8]. However, in the pooled analysis, 37% of patients initially managed non-operatively underwent appendectomy within one year for either primary failure of non-operative management or subsequent recurrence of appendicitis. Based on careful examination of this meta-analysis and its associated studies, the results of these studies need to be carefully extrapolated to pediatric practice because they included very few children. Data on non-operative management in children was limited to one retrospective study, conducted outside the United States, which demonstrated an 81% success rate of non-operative management in patients with suspected uncomplicated appendicitis at one year follow-up [1].

In addition, most of the adult studies did not select for patients with suspected uncomplicated appendicitis; these studies enrolled all patients with appendicitis regardless of duration of symptoms, imaging findings or suspicion of more advanced or complicated disease. Based on these studies, specific clinical factors associated with a higher likelihood of failure of non-operative management include the presence of a fecalith or fluid collection on imaging and the presence of symptoms for >48 hours.

A prospective clinical trial from NCH has to date confirmed the effectiveness of a non-operative treatment strategy in children with suspected uncomplicated appendicitis ([4]). In this prospective non-randomized clinical trial, patients diagnosed with suspected uncomplicated appendicitis are allowed to choose between non-operative management and appendectomy. This study has enrolled 102 patients, 37 of whom selected non-operative management. The overall success rate of non-operative management is 76% at a 1 year follow-up. In addition, patients in the non-operative group have demonstrated significantly faster return to normal activity and reported higher quality of life scores. Also, consistent with the adult studies, there has been no increase in the rates of complicated appendicitis (non-operative management group 3% vs. surgery group 11%) or overall complication in patients choosing non-operative management compared to patients choosing appendectomy. The results of this study confirm that non-operative management is a reasonable treatment strategy for children with uncomplicated appendicitis. At NCH, non-operative management has been incorporated into the standard treatment protocol for uncomplicated acute appendicitis (See Appendix).

This proposed study will expand upon the previous NCH study to perform a multi-institutional study to determine the generalizability of a non-operative management strategy for uncomplicated appendicitis across ten Children’s Hospitals that comprise the Midwest Pediatric Surgical Consortium. This multi-intuitional study will allow us to: 1) determine the success rate of non-operative management; 2) compare differences in disability days, complication rates, and quality of life between patients choosing non-operative management and those choosing appendectomy; and 3) determine the cost-effectiveness of a non-operative management strategy for suspected uncomplicated appendicitis. Successful completion of this study may result in a paradigm shift away from operation, thereby decreasing the number of appendectomies performed and reducing the overall costs and risks associated with pediatric appendicitis.

1.3 Compliance Statement

This study will be conducted in full accordance of all applicable institution’s Research Policies and Procedures, the bylaws of the Midwest Pediatric Surgical Consortium, and all applicable

federal and state laws and regulations including the HIPAA Privacy Rule. Any episode of non-compliance will be documented.

The investigators will perform the study in accordance with this protocol and will report unexpected problems in accordance with each institution's IRB Policies and Procedures and all federal requirements. Collection, recording, and reporting of data will be accurate and will ensure the privacy, health, and welfare of research subjects during and after the study. In addition, site principal investigators will report all unexpected problems and adverse events to the study principal investigator (PI) and research team at NCH (overall study PI and data coordinating center). Finally, all events will be reviewed by the study Data Safety and Monitoring Committee (DSMC) every six months throughout the period.

2 STUDY OBJECTIVES

2.1 Primary Objectives

1) To compare differences in treatment-related disability days at 1 year between non-operative management and surgery in children with uncomplicated appendicitis across 10 children's hospitals.
2) To determine the 1 year success rate of non-operative management of uncomplicated appendicitis at 10 children's hospitals

2.2 Secondary Objectives

1) To compare rates of complicated appendicitis between groups at 30 days, and at one, two, and three years.
2) To determine differences in disability days at 30 days which includes the inpatient days, days of missed school, days until return to normal activities, days until guardian returns to normal schedule, and days for doctor or ER visits.
3) To compare quality of life measures at 30 days and at one year between the treatment groups.
4) To determine cost-effectiveness of non-operative management at one year, including initial admission, subsequent emergency department (ED) visits or physician visits, subsequent readmissions and reoperations and additional imaging related to appendicitis or complications of appendectomy.
5) To compare rates of treatment-related complications at 30 days, 6 months and at one, two, and three years.

3 INVESTIGATIONAL PLAN

3.1 Study Design

This is a prospective, non-randomized multi-institutional trial investigating a non-operative management strategy for children (7-17 years old) with uncomplicated appendicitis across children's hospitals. In this trial, patients and their families will choose between two treatment options: non-operative management with antibiotics alone or urgent appendectomy. There will be two groups: those choosing non-operative management (Non-Operative group) and those

choosing appendectomy (Surgery group). Outcomes will include determining the success rate of non-operative management and comparing differences in disability days, complication rates, cost of care, and quality of life between the two groups.

This is a patient choice trial rather than a randomized trial because we believe that the success of each treatment option depends on which outcomes are most important to the patient and their family [23-27]. For example, although initial non-operative management of early appendicitis may be safe for most patients, appendectomy may be a better treatment option for patients who live in remote areas or for families who are so fearful of a recurrence that they are likely to return to the ED every time their child develops abdominal pain. For these patients, the risk of post-operative complications may be perceived as minor compared to the benefit of a curative appendectomy. In contrast, for families who are averse to surgery, initial non-operative therapy may be the least stressful and most appealing choice because it may eliminate the need for an operation and its inherent risks while expediting return to activities. For these reasons, we opted to allow eligible patients and their families to choose between surgery and non-operative management rather than randomize patients to one of the two treatment groups.

3.2 Study Duration and Enrollment

3.2.1 Duration of Study and Enrollment

Enrollment for this study will be conducted through October 2018 with annual follow-up phone calls for subjects who choose antibiotic treatment until age 18 years and three years for subjects who choose surgery. A minimum of one year of data will be necessary to collect to assess our primary outcome.

3.2.2 Total Number of Subjects Projected and Site

The study will be coordinated through the Center for Surgical Outcomes Research (CSOR) within the Center for Innovation in Pediatric Practice (CIPP) at the Research Institute at Nationwide Children's Hospital (RINCH) in Columbus, OH. A total of 300 children are projected to be enrolled from our institution as part of a broader cohort of 1100 children with approximately 40% choosing non-operative management. We plan for 91 patients to be enrolled at each site but with larger sites (such as NCH) potentially enrolling more patients as needed to meet the necessary enrollment numbers (up to a maximum of 300 patients). Study participants will be recruited after surgical consultation has been performed in the ED and will be consented either in the ED or surgical inpatient units of each institution.

Follow-up research phone calls and surveys will be conducted (see schedule below) by study coordinators at each site through the first two weeks and then by a study coordinator at NCH for all subsequent phone calls through three years for those subject choosing surgery and annually for those who choose antibiotics. Patients will also follow up on an outpatient basis with each institution's department of surgery in accordance with standard of care.

3.3 Study Population

Children between the ages of 7-17 who are diagnosed with early appendicitis will be screened for eligibility.

3.3.1 Inclusion Criteria

- English and non-English speaking patients
- Age : 7-17 years
- US, CT, or MRI-confirmed early appendicitis:
    - US: hyperemia, ≤ 1.1 cm in diameter, compressible or non-compressible, no abscess, no fecalith, no phlegmon
    - CT or MRI: hyperemia, fat stranding, ≤ 1.1 cm in diameter, no abscess, no fecalith, no phlegmon
- WBC count > 5,000/µL and ≤ 18,000/µL
- Abdominal pain ≤ 48 hours prior to receiving antibiotics

3.3.2 Exclusion Criteria

- History of chronic intermittent abdominal pain
- Pain > 48 hours prior to first antibiotic dose
- Diffuse peritonitis
- Positive urine pregnancy test
- WBC ≤ 5,000/µL or ≥ 18,000/µL
- Presence of a fecalith on imaging
- Evidence on imaging studies for evolving perforated appendicitis, including abscess or phlegmon
- Communication difficulties (e.g. severe developmental delay)

4 STUDY PROCEDURES

4.1 Screening and Enrollment

During initial contact with a potential subject, a physician-member of the surgical consult team will assess their eligibility through satisfaction of both inclusion and exclusion criteria. If all eligibility criteria are met, a physician-member of the research team will invite the child and legal guardian to enroll. The physician-member of the research team will then review the written information about the study and answer any questions. The patient and family will then choose between non-operative management with antibiotics alone (Non-Operative group) and urgent appendectomy (Surgery group).

Upon enrollment, study staff at each institution will be contacted by the physician-member of the research team who obtained consent/assent. The study staff will conduct all data collection throughout the study, with study staff at each institution conducting data collection during the inpatient admission and through two weeks post-discharge. Study staff at NCH will conduct data collection after the two-week period. A research team call schedule will be maintained that assigns a physician-member of the NCH research team to be available for consultation with each institution's clinical team. The clinical team will also be able to contact a physician-member of the research team by pager 24 hours per day. This information will be provided to the clinicians caring for the patients and will be posted in pre-specified areas within the surgical wards. Study staff will also contact families with visit reminders via phone and letter, if necessary. Families in the non-operative group who do not respond to our follow-ups at six months, one year and/or the yearly follow-up will be considered lost to follow-up.

4.2 Study Procedures: Non-Operative Group

4.2.1 Hospital Course

4.2.1.1 Treatment Intervention

Upon enrollment, the subject will receive IV Zosyn (piperacillin-tazobactam 2 g/0.25 g)[1]. This is the standard therapy given to every patient diagnosed with appendicitis at NCH. Dispensation of the drug is based on weight. Participants <40 kg will be prescribed a dose of 300 mg piperacillin component/kg/day divided every eight hours and those >40 kg will receive Zosyn at a dose of 3.375 g piperacillin-tazobactam every six hours[2]. Patients with penicillin allergies will receive IV Cipro (ciprofloxacin) at a dose of 30 mg/kg/day divided every eight hours up to 1200 mg per day and IV Flagyl (metronidazole) at a dose of 30 mg/kg/day divided every six hours up to 500 mg per dose[3].

4.2.1.2 Treatment Algorithm

Vital signs and pain score will be assessed per standard nursing protocol[4] (please see appendix for sample pain scores used) for each institution. If the pain improves, then the child will be offered food.

Cross-over to appendectomy will occur in 2 situations:

1. Failure to improve after 24 hours of IV antibiotics: Patients who do not exhibit clinical improvement (decreased tenderness, resolution of fever) or do not report symptomatic relief (decreased pain, resolution of nausea/vomiting, advancement of diet) after receiving 24 hours of intravenous antibiotics will be recommended for appendectomy. If there is either objective or subjective improvement, then the child can continue on IV antibiotics for up to another 24 hours.
2. If clinical status worsens: If a patient's symptoms worsen (increased abdominal pain) or there is evolving objective evidence of systemic signs of infection (increasing tachycardia, hypotension, persistent fever, or decreased mental status), then he/she will be recommended for appendectomy.

Failure to improve or worsening of clinical status that leads to an appendectomy is considered treatment failure of non-operative management. This is different from an independent request from the parent or legal guardian for the patient to undergo surgery, despite clinical improvement and/or symptom relief, which is considered conversion to the surgery group or elective withdrawal from the study and not a treatment failure of non-operative management.

Patients whose symptoms begin to resolve (decreased abdominal pain, no nausea or vomiting, or fever resolution) and who tolerate regular food will be discharged. Patients will be sent home on

---

[1] IV Zosyn is the standard regimen for our institution. You may change this and the following directions on dosing to a different antibiotic based on your institutional policies and standard of care.
[2] IV Zosyn is the standard regimen for our institution. You may change this and the following directions on dosing to a different antibiotic based on your institutional policies and standard of care.
[3] IV Cipro and Flagyl is the standard regimen for our institution for patients with penicillin allergies. You may change this antibiotic selection and these directions on dosing based on your institutional policies and standard of care.
[4] We have included a sample of NCH nursing protocol in the appendix. Your nursing protocol may be different.

standard oral Augmentin (<14 years will receive 45 mg amoxicillin component/kg/day divided every 12 hours and 14 years and older will receive 875 mg amoxicillin component every 12 hours, using the 400 mg/5mL suspension or 875 mg tablet depending on patient preference for liquid or tablets) for a total antibiotic course of seven days. For those children who have penicillin allergies, the alternative will be oral Cipro (30 mg/kg/day divided every 12 hours to a maximum of 1.5 g/day) and Flagyl (30 mg/kg/day divided every six hours up to 500 mg per dose).

Before discharge, the patient and legal guardian will complete a quality of life (QOL) survey (PEDSQL Quality of Life Inventory: Child and Parent Report) that asks about the child's health, feelings, and social functioning in and out of school and Decisional Regret, Decisional Self Efficacy, Decisional Conflict Scale and HealthCare Satisfaction surveys. Additionally, the family will be informed that while on antibiotics, if the child is on oral contraceptives, the efficacy of oral contraceptives may be impaired. The child's primary care physician will be notified via direct mail and a letter placed within the electronic medical record.
In addition, at the Nationwide Children's Hospital site only, legal guardians will be asked to complete questionnaires to assess decision making processes and satisfaction with their chosen treatment course; surgical management or nonoperative (antibiotic) management of appendicitis. Furthermore, legal guardians will also be asked to complete the PEDSQL HealthCare Satisfaction-Generic Module to assess their satisfaction with the healthcare provided.

In addition, we will collect information from the medical record to document procedures and treatments received during the initial hospital stay. We will record the costs directly from hospital departments that provide patient services, including inpatient (e.g., ICU) and outpatient nursing departments, diagnostic departments (e.g., labs, imaging), and pharmacies. We will also document payments received from all payers regarding each relevant procedure (by reviewing medical charts and searching claims data) and track the time spent by surgeons and nurses spent on non-operative management.

When patients undergo a surgical or interventional radiology procedure related to treatment of appendicitis, such as an appendectomy or intra-abdominal drain placement, study staff will collect information from medical records and clinical interview with the patient and family to document procedures and treatments received during that additional clinical visit or hospital stay and any complications related to that procedure. Please see the form in the appendix (Data collection form for additional procedures) for data points to be collected as part of this process. Chart reviews will be conducted by each site at 1 year, 6 months to 6 weeks prior to the first analysis, and 3 years to validate patient outcomes.

We will also collect the demographic and socioeconomic information of both the patient and the legal guardian including: age, race, ethnicity, and gender of the patient; annual parent/guardian income range; guardian occupations; and specific insurance coverage status for the patient.

4.2.2 Follow-Up

4.2.2.1 2-5 days and 10-14 days

A member of each institution's research team will call the family for a follow-up. Any concerns, issues or complications regarding the oral antibiotics or the study will be discussed. In addition, how many days the child missed from normal activities, including school, gym, recess, sports, and

other after school activities, will be recorded. Similarly, the number of days that the legal guardian spent without a normal schedule will also be assessed. Please see the form in the appendix (Survey at 2-5 days and 10-14 days for Non-Operative group) for data points to be collected during this phone call. The family will also be asked if they have any comments regarding the study.

4.2.2.2 30 days (±10 days)

A member of the central research team from NCH will call or email the family for a follow-up. Please see the form in the appendix (Survey at 30 days, 6 months, 1 year, and annually for Non-Operative group) for data points to be collected during this phone call. The child and legal guardian will complete the same QOL survey (PEDSQL Quality of Life Inventory: Child and Parent Report) completed before hospital discharge either in person or over the phone.

The legal guardian will also complete a health satisfaction survey (PEDSQL Healthcare Satisfaction Generic Module: Parent Report) regarding their satisfaction with the care the child received at the hospital. We will also ask questions about any problems the child has had with their appendix since discharge, any other issues, and how many days the child missed their normal activities and the legal guardian could not engage in their normal schedule. We will ask specifically about subsequent ED visits or physician visits, readmissions and operations and additional imaging related to appendicitis. We will also ask about the out-of-pocket expenditures directly spent on post-treatment medical care and returning visits (e.g. insurance copayments; costs of drugs) and other expenditures related to the visits within 30 days. The family will also be asked if they have any comments regarding the study.

A VISA® ClinCard, preloaded with $25.00 and will be mailed to the legal guardian's address at the completion of the 30 day follow-up.

4.2.2.3 6 months (±20 days)

A member of the central research team from NCH will call or email the family and ask questions about any problems the child has had with their appendix since their 30-day follow-up. Specifically, we will ask about subsequent ED visits or physician visits, readmissions and operations, additional imaging, and missed days from normal activities by the patient and legal guardian that were related to appendicitis. We will also ask about the out-of-pocket expenditures directly spent on post-treatment medical care and returning visits (e.g. insurance copayments; costs of drugs) and other expenditures related to the visits within six months. Please see the form in the appendix (Survey at 30 days, six months, one year, and annually for Non-Operative group) for data points to be collected during this phone call. The family will also be asked if they have any comments regarding the study, $25.00 will be loaded onto the participant's VISA® Clincard upon completion of the 6 month follow-up.

4.2.2.4 1 year (±30 days)

A member of the central research team from NCH will call or email the family and ask questions about any problems the child has had with their appendix since their six-month follow-up. Specifically, the research team will ask about subsequent ED visits or physician visits, readmissions and operations, additional imaging, and missed days from normal activities by the patient and legal guardian that were related to appendicitis.

If previously consented to at the time of enrollment, a team member will contact the subject's PCP by phone or letter (with accompanying survey) as part of the 1 year follow-up to assess for events related to the diagnosis, treatment, and health care services provided for the subject's appendicitis treatment during the past year. A specific section of the consent form asks the patient's legal guardian to either accept or decline this part of the study.

Subjects who will turn 18 before their one-year follow-up will be asked to sign the informed consent used for the study. Consent will either be obtained by a member of the local research team or the NCH research team. After speaking with the subject and explaining the study, an informed consent will be mailed to the subjects to sign so that the research team may continue to collect data up to one year after their initial hospital discharge.

We will also ask about the out-of-pocket expenditures directly spent on post-treatment medical care and returning visits (e.g. insurance copayments; costs of drugs) and other expenditures related to the visits within one year. Please see the form in the appendix (Survey at 30 days, six months, one year, and annually for Non-Operative group) for data points to be collected during this phone call. The family will also be asked if they have any comments regarding the study.

$50.00 will be loaded onto the participant's VISA® Clincard upon completion of the 1 year follow-up.

For those 1 year follow-ups that are not completed, we will conduct one final phone call and send a letter outside the window in an attempt to obtain data for final follow-up. If we are unable to reach the patient, then the local site will also try one final phone call

4.2.2.5 Annually (±30 days) through the age of 18

A member of the central research team from NCH will call or email the family once a year until three years after initial treatment. The same follow-up questions as those asked during the one year follow-up will be asked. Please see the form in the appendix (Survey at 30 days, six months, one year, and annually for Non-Operative Group) for data points to be collected during this phone call. In addition, we will call all patients and families for their consent to follow them with an annual phone call or email until their child turns 18 years of age. The family will also be asked if they have any comments regarding the study.

4.3 Study Procedures: Surgery Group

4.3.1 Hospital Course

4.3.1.1 Treatment Intervention

Each institution's standard of care for managing appendicitis will be applied.

Before discharge, the patient and legal guardian will complete a QOL survey (PEDSQL Quality of Life Inventory: Child and Parent Report) that asks about the child's health, feelings, and social functioning in and out of school.

In addition, we will collect information from the medical record to document procedures and treatments received during the initial hospital stay. We will record the costs directly from hospital departments that provide patient services, including inpatient (e.g., ICU) and outpatient nursing departments, diagnostic departments (e.g., labs, imaging), and pharmacies. We will also

document payments received from all payers regarding each relevant procedure (by reviewing medical charts and searching claims data) and track the time spent by surgeons and nurses spent on non-operative management.

We will also collect the demographic and socioeconomic information of both the patient and the legal guardian including: age, race, ethnicity, gender, annual parent/guardian income range, guardian occupations and specific insurance coverage status.

4.3.2 Follow-Up

4.3.2.1 30 days (±10 days)

A member of the central research team from NCH will call or email the family for a follow-up. The child and legal guardian will complete the same QOL survey (PEDSQL Quality of Life Inventory: Child and Parent Report) completed before hospital discharge either in person or over the phone. Please see the form in the appendix (Survey at 30 days, six months, one year, and annually for Surgery group) for data points to be collected during this phone call.

The legal guardian will also complete a healthcare satisfaction survey (PEDSQL Healthcare Satisfaction Generic Module: Parent Report) regarding their satisfaction with the care the child received at the hospital. We will also ask questions about any problems the child has had with their appendix since discharge, any other issues, and how many days the child missed normal activities including school, gym, recess, sports, and other after school activities will be recorded. Similarly, the number of days the legal guardian could not engage in their normal schedule will be recorded. We will ask specifically about subsequent ED visits or physician visits, readmissions and operations and additional imaging related to their appendectomy. We will also ask the out-of-pocket expenditures directly spent on post-treatment medical care and returning visits (e.g. insurance copayments; costs of drugs) and other expenditures related to the visits within 30 days. The family will also be asked if they have any comments regarding the study.

A VISA® ClinCard, preloaded with $25.00 and will be mailed to the legal guardian’s address at the completion of the 30 day follow-up.

4.3.2.2 6 months (±20 days)

A member of the central research team from NCH will call the family and ask questions about any problems the child has had with their appendix since their 30-day follow-up. Specifically, we will ask about subsequent ED visits or physician visits, readmissions and operations, additional imaging, and missed days from normal activities by the patient and legal guardian that were related to their appendectomy. We will also ask about the out-of-pocket expenditures directly spent on post-treatment medical care and returning visits (e.g. insurance copayments; costs of drugs) and other expenditures related to the visits (e.g. transportation costs) within one year. Please see the form in the appendix (Survey at 30 days, six months, one year, and annually for Surgery group) for data points to be collected during this phone call. The family will also be asked if they have any comments regarding the study.

$25.00 will be loaded onto the participant’s VISA® Clincard upon completion of the 6 month follow-up.

4.3.2.3 1 year (30 days)

A member of the central research team from NCH will call or email the family and ask questions about any problems the child has had with their appendix since their six-month follow-up. Specifically, we will ask about subsequent ED visits or physician visits, readmissions and operations, additional imaging, and missed days from normal activities by the patient and legal guardian that were related to their appendectomy. We will also ask about the out-of-pocket expenditures directly spent on post-treatment medical care and returning visits (e.g. insurance copayments; costs of drugs) and other expenditures related to the visits (e.g. transportation costs) within one year. Please see the form in the appendix (Survey at 30 days, six months, one year, and annually for Surgery Group) for data points to be collected during this phone call.

For subjects who will turn 18 before their one-year follow-up, they will be asked to sign the informed consent used for the study. After speaking with the subject and explaining the study, an informed consent will be mailed to the subjects to sign so that we may continue to collect data up to 1 year after their initial hospital discharge. The family will also be asked if they have any comments regarding the study.

$50.00 will be loaded onto the participant's VISA® Clincard upon completion of the 1 year follow-up.

For those 1 year follow-ups that are not completed, we will conduct one final phone call and send a letter outside the window in an attempt to obtain data for final follow-up . If we are unable to reach the patient, then the local site will also try one final phone call.

4.3.2.4 Annually (±30 days) through age of 18

A member of the research team will call the family once a year until three years after initial treatment. The same follow-up questions as those asked during the one year follow-up will be asked. Please see the form in the appendix (Survey at 30 days, six months, one year, and annually for Surgery Group) for data points to be collected during this phone call. In addition, we will call all patients and families for their consent to follow them with an annual phone call or until their child turns 18 years of age. The family will also be asked if they have any comments regarding the study.

5 STATISTICAL CONSIDERATIONS

5.1 Primary and Secondary Endpoints

The primary endpoints are: 1) to compare differences in treatment-related disability days at 1 year between non-operative management and surgery in children with uncomplicated appendicitis across 10 children's hospitals; and 2) to determine the 1 year success rate of non-operative management of uncomplicated appendicitis at 10 children's hospitals. Secondary endpoints that will be compared between groups are: rates of complicated appendicitis between groups at 30 days, one year, two years and three years; differences in disability days at 30 days and one year (which includes the inpatient days, days of missed school, days until return to normal activities, and days until guardian returns to normal schedule and days for doctor or ER visits); quality of life measures at discharge, 30 days, and

one year; cost-effectiveness of non-operative management at one year (including initial admission, subsequent ED visits or physician visits, subsequent readmissions and reoperations and additional imaging related to appendicitis or complications of appendectomy); and rates of treatment related complications at 30 days, one year, two years, and three years. A summary of the outcomes that will be assessed are listed in the table below.

Table: Outcomes to be assessed

Outcome	Time points
In all patients	
Decisional Regret***	Index hospitalization
Decisional Self Efficacy***	Index hospitalization
Decisional Conflict Scale ***	Index hospitalization
HealthCare Satisfaction ***	Index hospitalizaton
Disability Days	30 days, 1 year
Length of Stay	Index hospitalization
Emergency Department Visits	30 days, 1 year, 2 year, 3 year
Readmissions	30 days, 1 year, 2 year, 3 year
Complicated Appendicitis	30 days, 1 year, 2 year, 3 year
Post-treatment Related Complications	30 days, 1 year, 2 year, 3 year
Satisfaction with Health Care	30 days
Health Related Quality of Life	Index hospitalization, 30 days, 1 year
Satisfaction with Decision	30 days, 1 year
Health Care Associated Costs	30 days, 1 year
Incremental Cost Effectiveness	1 year
Additional surgical or interventional procedures	30 days, 1 year, 2 year, 3 year
In Operative Subgroup only	
Postoperative Infections	30 days
Re-operation	30 days, 1 year, 2 year, 3 year
In Non-operative Subgroup only	
Success rate	30 days,1 year, 2 year, 3 year
Need for appendectomy during initial admission	Index hospitalization
Recurrence	30 days, annually
Antibiotic complications	30 days

5.2 Statistical Methods

All patient baseline demographics and clinical characteristics will be described and summarized overall and between treatment groups. The balance/imbalance of these characteristics will be studied and reported, particularly for analyses comparing the two treatment groups. Balance in all pre-treatment characteristics between groups will be measured through the standardized difference of each covariate. All of these covariates (including demographics, race, ethnicity, socioeconomic variables, clinical and imaging characteristics, laboratory values and patient recruitment site) will be collected from the patient, caregiver and medical record at the time of enrollment; this will allow robust data capture with minimal missing data.All measured pre-

treatment covariates considered to potentially confound the relationship between treatment and outcome or those considered to be highly associated with outcome will be included in the development of the propensity score/probability of treatment model.

Missing Data: Missing data will be minimized in several ways. First, data on all demographics and clinical characteristics will be collected at the time of enrollment which should minimize missing data for covariates necessary for stratification and sensitivity analyses. Data management will be monitored weekly by central study staff to ensure complete data entry on all enrolled patients within the REDCap database. Missing elements will be identified and relayed to each site weekly to allow for remediation and prevention of future instances of missing data. Patient incentives will be used to minimize missing outcome data due to patient dropout and short, simple language follow-up questions and surveys have been chosen to minimize the chances that patients will fail to provide data due to the time burden of the surveys or not understanding the questions.

If a participant drops out, we will document the reason for dropping out, whose decision it was (patient, family member, physician), drop out date, and whether it was a complete or partial drop out. We will attempt to collect information on success rate of non-operative management using medical records unless consent is withdrawn. All participants' information/data will be used in all the study analyses. Patients who are lost to follow-up or withdraw from the study will be censored from longitudinal analyses after the date in which they were lost to follow-up or withdrew.

Any outliers and/or missing data will be carefully revised and addressed by going back to the original sources to double check for potential errors. The amount of missing data will be reported and if patterns of missing data are detected, the statisticians will carefully evaluate them. Sensitivity analyses will be performed to address the effect of missing data on the primary and secondary endpoints. The amount of missing data could be indicative of problems with the design or feasibility of the study. Therefore, if more than 15% of data are missing, the generalizability of the study will be limited and study outcomes may be inconclusive. We will report differences between groups with and without missing data as part of these analyses including examining differences across sites, by treatment choice, by race, ethnicity, gender, SES status, and distance from the treating institution. We will consider multiple imputation exclusively for the purpose of sensitivity analyses.

Analysis Methods: Analysis of the primary endpoint of disability days of the child will employ propensity score methods as a means to quantify differences in baseline characteristic between groups and to balance them in final analysis. We will utilize inverse probability weighting with the estimated propensity score and will consider further adjustment through regression models for any covariate that does not appear to be adequately balanced following estimation of the propensity score (incorporating the inverse probability weights). The distribution of the propensity scores (and inverse probability weights) will be described and graphically displayed. Stabilized inverse probability weights will be considered to mitigate the influence of very small estimated probabilities from the propensity score model. Regression models will be utilized to make inference, taking into account the estimated inverse probability of treatment weight and potential covariates, and will estimate standard errors either as robust standard errors or through bootstrap procedures. Inverse probability weighting was chosen instead of propensity score matching because it enables us to estimate the average treatment effect (ATE), rather than the average treatment effect in the treated (ATT), on all outcomes, including success of non-operative management. Propensity score weighting has been shown to

balance measured covariates and reduce bias in estimated treatment effects to a similar degree as propensity score matching in both empirical and simulation studies. Where possible, we will estimate effects stratified by institution and will examine the sensitivity of results to varying methods of analysis. Heterogeneity of effects due to three main factors of interest: age group (≤10 vs. >10 yrs), household income (<$50,000, ≥$50,000), transfer status, will be explored by evaluating these factors as potential effect modifiers by including each in a model to include the main treatment effect, the main factor effect and the interaction term for the treatment by factor. Treatment effects will be estimated for each level of factor and compared across these groups. Identification of effect modification will be made through tests of interaction in these models, which control the family-wise error rate of each of these 6 comparisons at the 2% level (translating to a maximum family wise error rate of 12%)

Analysis of the primary endpoint of success rate of non-operative management, defined as the success rate of non-operative management at 1 year, will be estimated and reported along with the 95% confidence interval. This rate will be examined overall and by treatment institution. The standardized success rate (to the overall population) will also be estimated and reported (along with the associated 95% CI). Success rate of non-operative management at 30 days and the rate of complicated appendicitis at 1 year (both secondary endpoints) will be estimated along with their 95% CI. These analyses are all hypothesis driven with an expected 1-year success rate of non-operative management of >75% across all 10 institutions with a range between 70-80% with no increase in the rate of complicated appendicitis. Both primary and secondary endpoint points will be examined stratified by various socio-economic (SES) and patient demographic characteristics, including race, ethnicity, household annual income and number of household residents. These are exploratory analyses.

Secondary outcomes that involve the comparison of treatment groups will employ propensity score methods, as a means to quantify differences in baseline characteristic between groups and to balance them in final analysis. Analysis will mimic that of the primary endpoint comparison above for disability days, including inverse probability weighting by the estimated propensity score and will consider further adjustment in regression models for any covariate that does not appear to be adequately balanced following estimation of the propensity score. Where possible, we will estimate effects stratified by institution and will examine the sensitivity of results to varying methods of analysis. These are hypothesis driven analyses (See “Primary and Secondary Endpoints” section above for specific definitions of each outcome) with expected: rates of complicated appendicitis to be similar; rates of treatment related complications to be lower in the non-operative group; lower caregiver disability days at 30 days and 1 year in non-operative group; higher HRQOL scores in non-operative group at 30 days, and similar HRQOL, health care satisfaction, satisfaction with decision scores at 1 year; longer hospital length of stay in the non-operative group; similar antibiotic associated complications; and similar rates of imaging related to appendicitis. We will pay specific consideration to the potential of varying effects of treatment by treatment site, SES and patient characteristics including age, household income and transfer status; these are exploratory analyses which will be carried through including interaction effects in the models and by fully stratifying analyses as described above.

We will explore the candidate instrumental variables of transfer status and distance from treating hospital. It is hypothesized that each of these impacts the treatment choice but does not directly influence the outcomes of interest. Sensitivity of results to model assumptions and bias in analytic methods, including un-measured confounding, will be explored for each endpoint of interest to assess the potential impact on inferences. An interim analysis will be performed to

asses for futility after 25% (n=91) of the total number of non-operative patients (n=363) to be enrolled has reached their one-year follow-up. Based on the first 227 participants (25% of 908), an initial set of inverse probability weights will be developed in order to assess initial futility of treatment. We will review participant balance following development of the initial selection into treatment model by IPW. Using IPW methods, we will assess the adjusted comparison of disability days between treatment groups. Further we will assess the success rate of the non-operative management group on these first 91 participants. At the interim analysis we will evaluate futility (Rejection of H1) of both primary endpoints through Lan-Demets spending function approach with O'Brien-Fleming boundary for each primary endpoint.

Throughout the trial, the success rate of non-operative management at 30 days and disability days of the child and the rate of complicated appendicitis at 1 year will be monitored for safety. These rates will be regularly reported to the Data and Safety Monitoring Committee (DSMC) and will be formally examined during the interim analysis assessment. The overall 30 day success rate is expected to be no lower than 80%. Further, if the rate of complicated appendicitis exceeds 30% in the patients who fail non-operative treatment and undergo appendectomy, this will be considered unacceptable. In the event that these rates (both primary and secondary) appear to be unacceptable, we will investigate if they vary by institution or patient characteristics.

Cost Effectiveness Analyses

The cost-effectiveness analysis is taken from a hospital perspective as well as from societal perspective. We will consider both the direct medical costs and indirect medical costs associated with informal care provided by parents. The direct medical costs are defined by the one year total health care expenditures per patient in our study, including that of initial admission, subsequent ED visits or physician visits, subsequent readmissions and reoperations, and additional pharmaceutical, imaging or laboratory procedures related to appendicitis or complications of appendectomy. The institutional claims data is augmented by that of the parental survey to obtain the cost information of patients who go to a separate hospital for subsequent care after the initial discharge. The follow-up surveys will also document the number of days the parents were absent from work due to the chosen appendicitis-related care for their child. The indirect medical costs associated with informal care are represented by the wage lost due to the absent days from work (even though parents may not have a real reduction in salaries). For parents that do not work, we will estimate the indirect costs based on the average income for their zip code and age groups. We will conduct both cost-saving analysis and cost-effectiveness analysis. The end point is whether a patient developed a complication related to their chosen treatment at 1 year follow-up; multiple complications may occur during the year. To analyze the cost-saving from non-operative management, the total costs (direct plus indirect) will be compared between the Non-operative and Surgery groups. For the cost effectiveness analysis, we will estimate the incremental cost-effectiveness ratios (ICERs), in terms of the marginal cost of an additional patient managed non-operatively who does not develop complications at 1 year. In addition, we will compute the marginal costs of an incremental unit of quality-of-life outcomes over 12 months derived from PEDSQL Quality of Life Inventory: Child and Parent Report. The health states used in this study are complication free or with complications at discharge, 30 days, 6 months or 12 months. The types of complications differ based on the chosen treatment with some specific to each treatment and some common to both. Common complications to both groups

include return to the ED or hospital readmission. Complications unique to non-operative management include a failure of non-operative management requiring appendectomy either in the hospital or after discharge and antibiotic associated side effects. Complications unique to appendectomy include complications associated with general anesthesia, bleeding or other intra-operative complications including inadvertent organ damage, wound complications, post-operative intra-abdominal infections, and repeat admission or operation for post-operative bowel obstruction due to scar tissue. Patients who fail non-operative management and undergo appendectomy are subsequently at risk for the complications related to appendectomy listed above. All analyses will be performed using decision tree analysis, with Data Pro HealthCare software (TreeAge Software, Inc., Williamstown, Massachusetts). In addition to the static probabilities and utilities (costs) derived from our clinical trial, we will also perform Monte Carlo sensitivity analysis using simulations at 10,000 trials each.

5.3 Sample Size and Power

The sample size needed to assess the primary outcome of the 1-year success rate of non-operative management is based on preliminary data that the expected point estimate is between 76-78%. Based on previous studies and input from our stakeholders and participating surgeons, the lowest acceptable success rate of non-operative management required for it to be considered as part of routine clinical practice would be 70% (p0) at 1 year follow-up. It is expected that the study success rate (p1) will be higher, ranging from 76-78%. Under a group sequential design, with one interim and one final analysis, overall type I error (two-sided) of 5 % (adjusted for the 2 primary endpoints), the maximum sample sizes required are listed below.

Given these estimates, the expected rates of patient choice of non-operative management (40%) or surgery (60%), and an approximately 10% expected rate of loss to follow up, we will enroll 908 patients. This sample size will provide more than adequate power to assess the other primary endpoint of disability days between groups. Based on previous data, we expect children who undergo non-operative management to have at least five fewer disability days in the year following treatment (assuming 10 days (sd=9.8) on average) than children who have initial surgery (assuming 15 (sd=7.7) on average). With the proposed sample sizes in each group, we will have >90% power to detect these differences, assuming an adjusted overall type I error of 5% (adjusted to account for the two primary endpoints).

Stratification by institution will be pursued to examine both primary and secondary endpoints and will be detailed descriptively. Heterogeneity of treatment effects will be formally explored for three patient demographic characteristics (age, household income, and transfer status). Comparison between groups will employ propensity score methods to quantify and adjust for differences in baseline characteristics and to balance them in final analyses, focusing on inverse probability weighting (marginal structural models).[28-30] We will consider further adjustment through regression models for any covariate that does not appear to be adequately balanced following propensity score model development and to assess potential effect modification (See Figure: Conceptual Model).

Figure: Conceptual Model

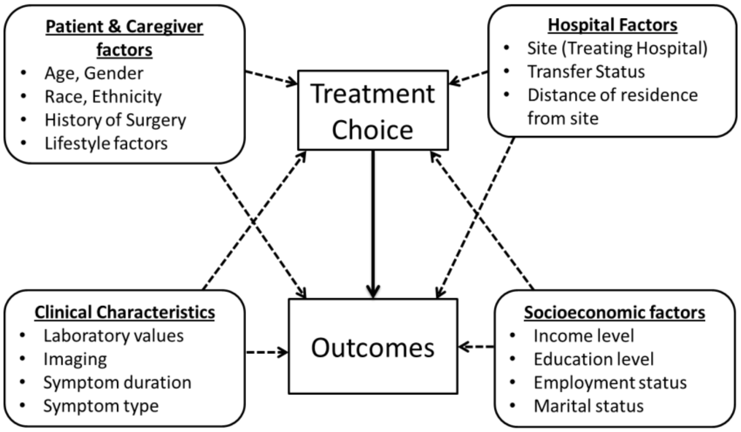

Table: Estimated required sample sizes of the non-operative group

Success rate	Power	Sample Size Non-operative group *
0.78	80%	250
0.77	80%	330

*Accounts for 1 interim analysis to determine futility with overall two-sided type I error rate of 5%.

6 STUDY ADMINISTRATION

6.1 Data Collection and Management

All clinical monitoring performed as standard procedure in the hospital, such as vital signs, pain scoring, dietary advancement, and other pertinent variables for the purposes of the study, will be recorded by local study staff in the study's central REDCap database maintained at NCH. NCH study staff will collect, compile and manage follow-up data. This data will be compiled on REDCap, a network software suite that provides encryption and password protection for health information, to which only study staff will have access.

Privacy and security will be maintained by minimizing the amount of identifiable data as much as possible. All information will be stored and managed on REDCap, and identifiable patient information not necessary for data analysis will not be exported from REDCap. Local data will be maintained on computers located at each institution's facilities that are maintained on a secure network. All necessary identifiers will be used only to identify the relevant source documents. Identified data will be shared with investigators at NCH (Principal Investigator: Dr. Peter Minneci, Site PI: Katherine Deans), and study staff at NCH that contact the patients at all sites by phone for follow-up phone interviews as detailed in our study design. Following completion of follow-up, the information will be de-identified once all of the data has been collected.

6.2 Confidentiality

All data and records generated during this study will remain confidential. All documents will be used solely for the use of this study by approved personnel. Consent forms and written surveys will be maintained at each institution in locked cabinets and all other data will be maintained in a central REDcap database that would be housed at NCH for the duration of the study. De-identified data exported from REDCap for statistical analysis will be maintained in password-protected files and on password-protected computers at NCH.

6.3 Regulatory and Ethical Considerations

6.3.1 Risk Assessment

The Division of Surgery at each participating institution has reviewed the results of the trials of non-operative management of appendicitis, including the recent pediatric study from NCH, and has determined that the evidence supports offering non-operative management as an alternative therapy to appendectomy as part of the routine care of patients with suspected uncomplicated appendicitis without a fecalith on imaging. Therefore, the following discussion of risks and benefits are divided into two sections: the risks associated with participating in the study to allow for data collection for research purposes, and the risks associated with each treatment option they will be offered as part of routine care for uncomplicated appendicitis and not directly related to study participation.

6.3.1.1 Risks associated with study participation

The quality of life surveys may make the subjects feel uncomfortable due to questions that ask about their emotional and social functioning. In addition, loss of confidentiality could be a risk.

6.3.1.2 Risks Associated with each treatment option (independent of study participation)

<u>Non-Operative Group</u>

Potential risks may include:

- No relief of symptoms (ex: persistent/prolonged pain) thereby requiring appendectomy, which will involve the inherent risks of surgery
- Possibility of experiencing interval progression of appendicitis, requiring appendectomy and may endure a prolonged hospital stay
- Recurrence of appendicitis at a later time
- Antibiotic associated side effects (see below)

Those who choose non-operative management will receive oral antibiotics (Augmentin[5], or if they have a penicillin allergy, Cipro and Flagyl[6]) on discharge. These antibiotics are no different than what is commonly used for pediatric intra-abdominal infections such as perforated appendicitis, Crohn's disease, and intra-abdominal abscesses. Some common side effects of

[5] PO Augmentin is the antibiotic we have selected as a department for this trial. It is the antibiotic we use for patients with ruptured appendicitis. You may change this based on your institutional and departmental policies.

[6] PO Cipro and Flagyl is the antibiotic we have selected as a department for this trial. It is the antibiotic we use for patients with ruptured appendicitis and a penicillin allergy. You may change this based on your institutional and departmental policies.

antibiotics[7] include: nausea, vomiting and diarrhea. Other rare side effects can include dizziness, skin rashes, drowsiness, metallic taste, difficulty breathing, and joint pain. While on antibiotics, the efficacy of oral contraceptives may be impaired.

Surgery Group

Potential risks may include:

- Side effects and rare complications associated with general anesthesia
- Wound complications including infections
- Bleeding or other intra-operative complication including inadvertent organ damage
- Post-operative intra-abdominal infections
- Repeat admission or operation for post-operative bowel obstruction due to scar tissue

6.3.2 Potential Benefits of Trial Participation

6.3.2.1 Benefits associated with study participation

Beyond the $25 and $50 Visa gift card incentives, there is no personal benefit to participating in the study, but information obtained may help others.

6.3.2.2 Benefits associated with each treatment option (independent of study participation)

Non-Operative Group

Potential benefits may include:

- Faster relief of symptoms (ex: resolution of pain),
- Shorter recovery period
- Avoiding risks of surgical complications

Surgery Group

Potential benefits may include:

- Appendicitis will never comeback
- No antibiotics after surgery

6.3.3 Risk-Benefit Assessment/Risk Minimization

6.3.3.1 Related to study participation

Regarding the quality of life surveys, participants have the option of skipping questions they find to be uncomfortable or simply wish to refuse answering. This will be stated on the informed consent.

Risk of loss of confidentiality will be minimized by using study ID numbers, limiting the number of team members with access to data with PHI and using a password protected database (REDCap) and password protected files.

[7] If you select different antibiotics, do note that they may have different side effects which should be noted here.

6.3.3.2 Related to Clinical care and specific treatment choice

<u>Non-Operative Group</u>

As part of routine care, patients will be closely monitored throughout their hospital stay by physicians and clinical staff to assess any adverse events and clinical symptoms. Patients who do not exhibit clinical improvement or report symptomatic relief as defined in our study procedure after receiving 24 hours of intravenous antibiotics will be recommended for appendectomy. Also, if a patient's symptoms worsen or there is evolving objective evidence of systemic signs of infection as defined in our study procedure, then he/she will be recommended for appendectomy.

Before discharge, patients and their caregivers will be given clear instructions on taking oral antibiotics and for reporting any concerns while on antibiotics. A research phone number for study staff at NCH will be provided in the informed consent should they have any questions or concerns relating to the study, and local contact information for medical questions will be provided consistent with the standard of care in surgical discharges. A magnetic reminder card with times for follow-up phones calls will be provided to each patient at discharge.

Regarding the quality of life surveys, participants have the option of skipping questions they find to be uncomfortable or simply wish to refuse answering. This will be stated on the informed consent.

6.3.3.3 Surgery Group

Patients will receive and be monitored according to the standard of care. Any adverse events will be handled according to hospital procedure. A magnetic reminder card with times for follow-up phones calls will be provided to each patient at discharge.

Regarding the quality of life surveys, participants have the option of skipping questions they find to be uncomfortable or simply wish to refuse answering. This will be stated on the informed consent.

6.3.4 Data Safety and Monitoring

The principal investigators, attending surgeons or fellows and clinical staff on the floor will be monitoring the data of participants including clinical symptoms and disposition at time of initiation onto the study and throughout their hospital stay. Vital signs, physical exam and pain scores will be assessed per each institution's nursing protocol and surgical service standards. An attending level surgeon will round on all patients every day to ensure that the child's clinical status is assessed. A research team call schedule will be maintained that assigns a member of the local research team to be available to check in with the clinical team daily. This person is also available by pager 24 hours per day. This information will be provided to the clinicians caring for the patients and will be posted in pre-specified areas within the surgical wards. Any suggestion of an adverse event identified by this person will be discussed with the principal investigator immediately.

Data will be monitored by research team members once a week. Research team members will ensure that all data (clinical data and questionnaires) collected are correctly completed. In addition, a Data Safety and Monitoring Committee (DSMC) has been formed and will meet every six months throughout the period during which patients are being recruited and

experiencing their first year of follow-up. The DSMC will review data provided by the primary study statisticians (Drs. Erinn Hade and Soledad Fernandez) and other study staff involved in data management and analysis. Dr. Martin Blakely (pediatric surgeon, Vanderbilt) will act as the chairman of the DSMB alongside Dr. William Garner, PhD (Statistician/Researcher, Children's Hospital of Eastern Ontario) and Dr. Adam Goldin (pediatric surgeon, Seattle Children's Hospital). In addition, the principal investigator and research team at NCH (overall study PI and data coordinating center) will be made aware of all adverse events as they occur and a quarterly conference call with all site PIs and the NCH PI and team will be held to review and discuss all AEs that occur in the trial. As mentioned above, all unexpected non-serious adverse events and serious adverse events relating to participation in the study will be reported verbally and in writing to the local IRB and the study PI and NCH IRB. The verbal report will occur within 48 hours of the occurrence. The written report of the serious adverse event (e.g., death or life-threatening adverse event) will be reported within seven days.

6.3.5 Adverse events (AE) and Serious adverse events (SAEs)

In the event that a child treated non-operatively should exhibit progression of their disease process to perforated appendicitis, which will be determined at the time of surgery, this will be considered an anticipated AE with an expected rate of 5% in the entire group of patients managed non-operatively and less than 30% in the subgroup of patients managed non-operatively who undergo appendectomy. In addition, patients treated non-operatively may experience an anticipated AE following discharge such as an unfavorable, non-life threatening reaction to the antibiotics. These include nausea, vomiting, diarrhea, dizziness, drowsiness, metallic taste, difficulty breathing, joint pain and also possible cutaneous reactions such as urticaria (skin rashes).

Patients who fail non-operative management are not expected to have an increased rate or severity of AEs following surgery. In essence, the patient who fails non-operative management initially will receive the same treatment as those patients in the Surgery Group.

For those patients in the Surgery Group, their potential anticipated adverse events will not differ from those of a patient who undergoes routine treatment for acute appendicitis. Specifically, the patient might experience an allergic reaction to the perioperative antibiotics given, discomfort and pain prior to and after the procedure, and standard complications associated with appendectomy. These complications include: bleeding; infection manifesting as either a superficial (cutaneous) or deep (intra-abdominal or pelvic) process; injury to adjacent structures including the small bowel, cecum, and right ureter; anesthesia-related complications; post-operative urinary tract infections and pneumonia; adhesive small bowel obstruction; and appendiceal stump leaks.

It is also possible that AEs might occur that are not directly related to the study. As such, all AEs will be classified as not related (clearly unrelated to study participation), possibly related (temporally related to study participation but could have been caused by other factors), or probably related (temporally related to study participation and cannot be reasonably explained by other factors) to study participation. The clinical study team will review all AEs as they occur and determine the seriousness and relatedness of them.

We do not expect any severe AE in either study group. The clinical study team will review SAE as they occur. All SAE deemed probably related to the study (and all deaths) will be reported to the local IRB and to the NCH IRB within 72 hours[8] of discovery and will be reviewed by the DSMC.

6.4 Recruitment Strategy

No active recruitment will take place. Subjects will be screened for eligibility once a diagnosis of suspected uncomplicated appendicitis has been determined and the surgical consult team has been contacted.

6.5 Informed Consent and Assent

All informed consents and assents will be performed by a physician-member of the research team.

If the child and legal guardian (for subjects < 18 years) are interested in participating in the study, a physician-member of the research team will guide the child and legal guardian through the informed consent/assent process. A one page decision aid will be made available if they seek extra information (see appendix). Written informed consent will be obtained from one legal guardian of subjects < 18 years of age. Written informed assent will be obtained from subjects ≥ 9 and < 18 years of age[9].

An informed consent and pros and cons script will be available to all consenting physician-members of the research team should they need it (see appendix).

Refusal to participate in the study will not affect the child's clinical care. Participants and their legal guardian have the right to switch to the standard treatment and/or withdraw from the study at any time. Withdrawal from the study will not affect receipt of clinical care.

6.6 Payment to Subjects and Families

The parent/legal guardian of all participants will be paid a $25 VISA® gift card after completion of their 30 day and 6 month follow-up. A $50 VISA® gift card will be paid after completion of their 1 year follow-up. This will be mailed to the legal guardian's address.

6.7 Confidentiality

Privacy and security will be maintained by minimizing the amount of identifiable data as much as possible. Only study identifications (IDs) will be used to identify patients on all data forms and all datasets used for analysis. The REDCap form linking study IDs to patient names and medical record numbers (MRNs) will be made available only to local study staff and will never be exported from REDCap or used during data analysis. All information will be compiled in REDCap, to which only study staff will have access.

---

[8] This is based on practice at NCH. Your institutional practice and IRB requirements may be different.

[9] This is based on our institutional requirements. Your institutional requirements may differ, but the trial should maintain this range for obtaining assent at a minimum. Note that at NCH, we are not required to secure assent for patients who are 8 years old; therefore, although patients may participate in this study if they are 8 years old, they do not have to sign assent forms.

APPENDIX: Example of Standard Nursing Protocol for Pain Assessment

Vital Signs & Pain Score

- Ordered Q4 hours as a standard on admission.
- Recorded by the RN or by the LPN/PCA if one is assigned – patients on any surgical service should have a pain score recorded Q4 hrs. and PRN.
- The score is documented by the RN or LPN.
- If an intervention is provided, its effectiveness should be reassessed within one hour with another pain score.

Vital Sign Protocol:

- You may skip ordered Q4hr vital signs at 4am when the following criteria are met:
    - Patient has been admitted for longer than 24 hours and is more than 24 hours past any surgical procedure
    - Patient has had stable vital signs for 24 hours (including being afebrile)
    - Patient must have had a complete set of midnight vitals including BP
- If the patient is on a pulse ox and/or cardiac monitor this data still needs to be documented at 4am.

Pain Scales used include FACES and VAS, demonstrated below:

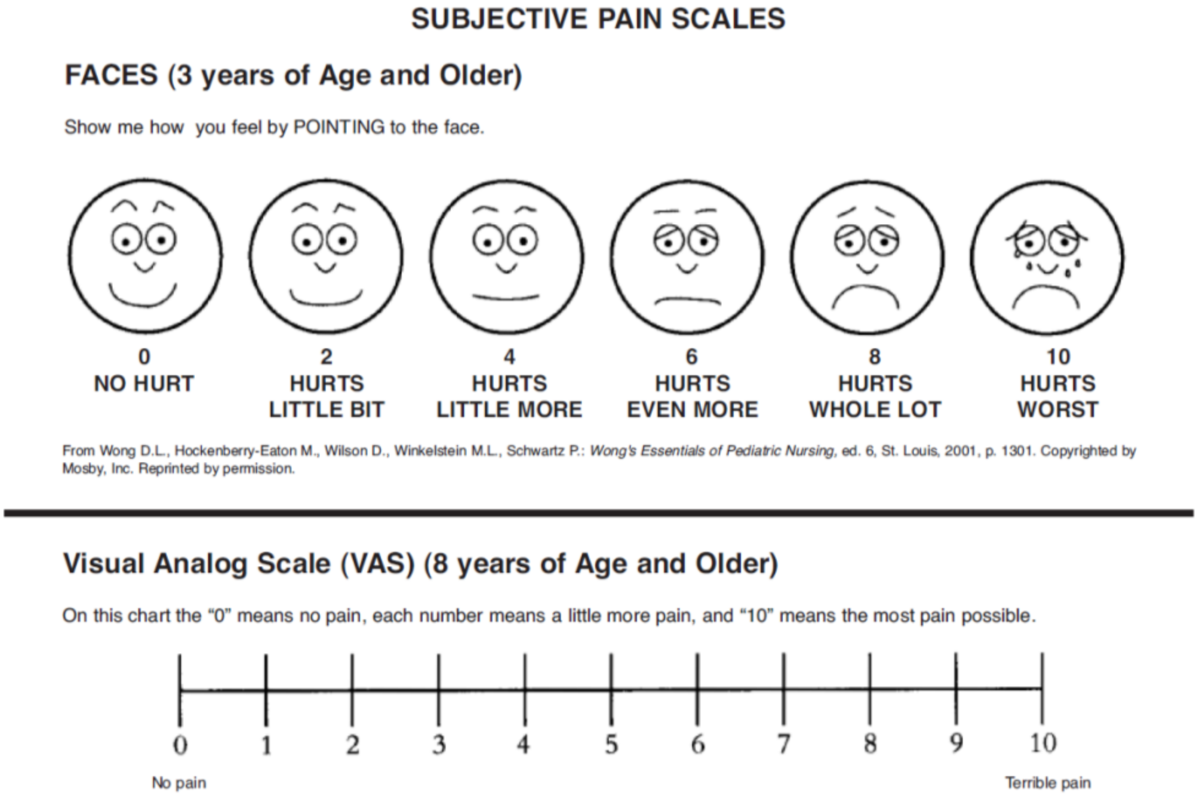

APPENDIX: Physician Informed Consent/Assent Script

Hi Mr./Mrs./Ms. ____________, my name is Dr. _________________.There is a research study in our department which involves treating appendicitis without surgery. Are you interested in learning more about it?

If "yes":

This study is being performed to determine if using antibiotics alone to treat appendicitis is as effective as performing surgery. The care and procedures performed here in the hospital would be the same as if you would be having surgery, without the surgical part. In addition, we would like you and (child's name) to fill out a quick survey before you leave the hospital, and over the phone at around 30 days after discharge. A member of the research team would also call you throughout the year and once a year after that to see how (child's name) is doing.

Are you interested in going through the informed consent?

You don't have to be in the study if you don't want to.

- Discuss informed consent and assent with legal guardian and patient.
- Show decision aid to legal guardian and patient.
- Leave room so that legal guardian and patient can privately discuss study and their decision.
- Come back and answer questions they may have.
- Remember to give the legal guardian a signed and dated copy of the Informed Consent and Assent

If "no" at any point:

Is it ok if you give us permission to track (child's name) treatment course and call you so that we may compare the surgical course to the non-operative course?

If "yes":

Like the non-operative group, we would like you and (child's name) to fill out a quick survey before you leave the hospital and by phone around 30 days after discharge. We will need to go through a different informed consent so that we can follow (child's name) treatment.

If "no":

Ok. Thank you for your time.

APPENDIX: Physician Pros and Cons Script

Hi Mr./Mrs./Ms. __, my name is Dr. __.There is a research study in our department which involves treating appendicitis without the need for surgery. Are you interested in learning more about it?

Discuss informed consent and assent with legal guardian and patient.
Show decision aid/informational sheet to legal guardian and patient, then also say:

The pros of non-operative management with antibiotics only include:

- Previous research studies show that it works in most adults and children with uncomplicated appendicitis
- Your pain may go away faster and you may recover sooner
- You may not need surgery – about 8 of 10 patients will not need an appendectomy

The possible cons of non-operative management with antibiotics only include:

- Your symptoms will not get better and you will need an appendectomy while in the hospital - this happens in 1 out of 10 patients
- Your appendicitis may come back in the future – this happens in another 1 out of ten patients
- Side effects of antibiotics
  - Most common: nausea (feeling sick), vomiting and diarrhea
  - Oral contraceptives may not work as well

The pros of surgery may include:

- Your child will never have appendicitis again.
- This is the most common way to treat appendicitis
- You can usually go home within 1 day after surgery

The possible cons of surgery may include:

- Your child will have some pain after surgery
- Most kids need several days of rest before going back to school and up to 2 weeks before returning to sports activities
- It will leave small scars on your belly
- There are risks associated with surgery - about 1 out of 10 kids will have some complication of surgery
- The most common are minor and include infections or problems with the wounds.
- Less common but more serious complications include
  - Infections inside the belly
  - Bleeding during surgery…
  - Injury to other organs near the appendix…
  - Scar tissue in the belly causing future blockage.

Remember to give the legal guardian a signed and dated copy of the Informed Consent and Assent

APPENDIX: Standardized Treatment Protocol for Uncomplicated Acute Appendicitis at Nationwide Children's Hospital

Standardized Treatment Protocol for Uncomplicated Acute Appendicitis at Nationwide Children's Hospital

Patients who meet the following criteria will be offered two treatment options: Appendectomy or antibiotics alone (Non-operative Management)

- Abdominal pain ≤48 hours
- Absence of peritonitis
- WBC <18k, >5k
- Ultrasound, CT, or MRI positive for appendicitis and
    - Diameter ≤1.1cm
    - No appendicolith
    - No evidence of perforation, abscess, or phlegmon

<u>Treatment Protocols:</u>

<u>Appendectomy:</u>

1. Admission, NPO, IV fluids
2. IV Zosyn (or Clinda/Gent)
3. To OR for urgent laparoscopic appendectomy
4. Post-operative management as per current protocols depending on intra-operative findings (simple vs. complicated)

<u>Antibiotics Alone (Non-operative management):</u>

1. Admission, IV fluids, NPO for minimum 12 hours and until improving
2. Serial observation and physical exams
    a) No improvement within first 24 hours or acute worsening triggers crossover to Surgery
    a) After 12 hour minimum NPO status, may advance diet (starting with clears) once improving
3. Antibiotic management:
    a) IV Zosyn (or Cipro/Flagyl) for minimum 24 hours
    b) Transition to PO Augmentin (or Cipro/Flagyl) once tolerating regular diet
    c) Discharge home to complete total course of 7 days of antibiotics with recommendation for yogurt intake
4. Please use standardized discharge instructions in Epic (.dinonopappendicitis)
5. Nurse phone follow up at 5-7 days and one month post-discharge:
   PLEASE EMAIL the following to Kim Welch at kimberly.welch@nationwidechildrens.org:
        Patient name
        MRN
        Date of discharge to

APPENDIX: Decision Aid: Pros and Cons of Choosing Surgery or Antibiotics-Only to Treat Appendicitis

Pros and Cons of Choosing Surgery or Antibiotics-Only to Treat Appendicitis

<u>What is appendicitis?</u>

When your appendix swells up, it's called appendicitis. Your appendix is a small pouch connected to your large intestine and has no known function. Anyone can get appendicitis. There isn't always a reason why appendicitis happens. Sometimes it happens after there is an infection in the intestine or something causes it to be blocked, then it gets swollen.

<u>Appendectomy (Surgery for Appendicitis)</u>

Pros:

- This is the usual way to treat appendicitis
- You will never have appendicitis again
- You can go home 1 to 2 days after surgery
- About 9 out of 10 children will not have a complication

Cons:

- You will be in some pain after surgery
- Most kids need a few days rest before going back to school and 1-2 weeks before returning to full activities
- It will leave 1-3 small scars on your belly
- There are some risks during surgery, such as bleeding or problems from the anesthesia
- Other possible risks include:
    - infection on the skin where you are cut, or inside your belly
    - staying longer at the hospital and needing more medicines like antibiotics
    - Needing additional surgery due to scars (adhesions) that can cause future blockage in your belly
- Side effects of antibiotics
    - Most common: nausea (feeling sick), vomiting and diarrhea

<u>Antibiotics Only</u>

Pros:

- Research studies show that it works in most adults and children
- Your pain may go away faster
- About 8 out of 10 children will not need surgery
    - No risks of surgery
- You may recover sooner

Cons:

- Your symptoms might not go away (ex: you are still in pain) and you will need an appendectomy which involve the risks of surgery
- Your appendicitis could come back in the future
- Side effects of antibiotics
    - Most common: nausea (feeling sick), vomiting and diarrhea
    - Oral contraceptives may not work as well

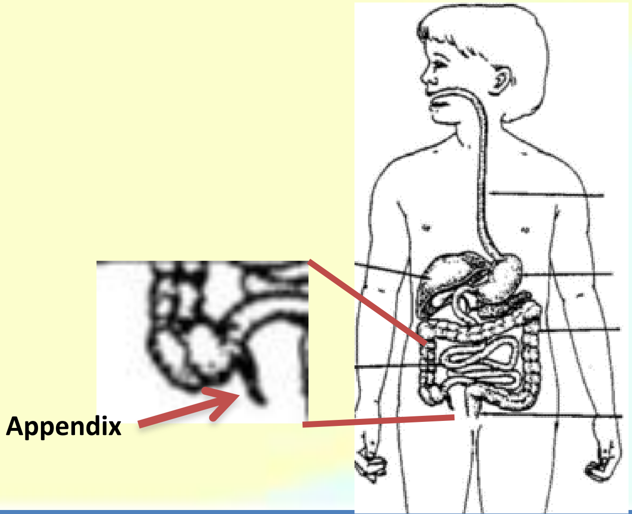

Picture from

APPENDIX: Survey at 2-5 days and 10-14 days for Non-Operative Group

APPENDIX: Survey at 30 days, 6 months, 1 year, and annually for Non-Operative Group

APPENDIX: Survey at 30 days, 6 months, and 1 year for Surgery Group

APPENDIX: Data collection form for patients undergoing surgery or IR procedures

Attached as separate documents

REFERENCES

1. Abes, M., B. Petik, and S. Kazil, *Nonoperative treatment of acute appendicitis in children.* J Pediatr Surg, 2007. **42**(8): p. 1439-42.
2. Eriksson, S. and L. Granstrom, *Randomized Controlled Trial of Appendectomy Versus Antibiotic-Therapy for Acute Appendicitis.* British Journal of Surgery, 1995. **82**(2): p. 166-169.
3. Fitzmaurice, G.J., et al., *Antibiotics versus appendectomy in the management of acute appendicitis: a review of the current evidence.* Can J Surg, 2011. **54**(5): p. 307-14.
4. Minneci, P.C., et al., *Feasibility of a Nonoperative Management Strategy for Uncomplicated Acute Appendicitis in Children.* J Am Coll Surg, 2014.
5. Hansson, J., et al., *Randomized clinical trial of antibiotic therapy versus appendicectomy as primary treatment of acute appendicitis in unselected patients (vol 96, pg 473, 2009).* British Journal of Surgery, 2009. **96**(7): p. 830-830.
6. Hansson, J., et al., *Antibiotics as First-line Therapy for Acute Appendicitis: Evidence for a Change in Clinical Practice.* World J Surg, 2012.
7. Styrud, J., et al., *Appendectomy versus antibiotic treatment in acute appendicitis. a prospective multicenter randomized controlled trial.* World J Surg, 2006. **30**(6): p. 1033-7.
8. Varadhan, K.K., K.R. Neal, and D.N. Lobo, *Safety and efficacy of antibiotics compared with appendicectomy for treatment of uncomplicated acute appendicitis: meta-analysis of randomised controlled trials.* BMJ, 2012. **344**: p. e2156.
9. Ein, S.H., J.C. Langer, and A. Daneman, *Nonoperative management of pediatric ruptured appendix with inflammatory mass or abscess: presence of an appendicolith predicts recurrent appendicitis.* J Pediatr Surg, 2005. **40**(10): p. 1612-5.
10. Ein, S.H., et al., *Is there a role for routine abdominal imaging in predicting postoperative intraabdominal abscess formation after appendectomy for pediatric ruptured appendix?* Pediatr Surg Int, 2008. **24**(3): p. 307-9.
11. Fraser, J.D., et al., *A complete course of intravenous antibiotics vs a combination of intravenous and oral antibiotics for perforated appendicitis in children: a prospective, randomized trial.* J Pediatr Surg, 2010. **45**(6): p. 1198-202.
12. Ein, S.H. and B. Shandling, *Is interval appendectomy necessary after rupture of an appendiceal mass?* J Pediatr Surg, 1996. **31**(6): p. 849-50.
13. Gillick, J., et al., *Laparoscopic appendectomy after conservative management of appendix mass.* Pediatr Surg Int, 2008. **24**(3): p. 299-301.
14. Hoffmann, J., A. Lindhard, and H.E. Jensen, *Appendix mass: conservative management without interval appendectomy.* Am J Surg, 1984. **148**(3): p. 379-82.
15. Puapong, D., et al., *Routine interval appendectomy in children is not indicated.* J Pediatr Surg, 2007. **42**(9): p. 1500-3.
16. Coran, A.G., et al., *Pediatric Surgery*. 7th Edition ed2012, Philadelphia: Elsevier Inc.
17. Zwintscher, N.P., et al., *Laparoscopy utilization and outcomes for appendicitis in small children.* J Pediatr Surg, 2013. **48**(9): p. 1941-5.
18. Lee, S.L., A. Yaghoubian, and A. Kaji, *Laparoscopic vs open appendectomy in children: outcomes comparison based on age, sex, and perforation status.* Archives of Surgery, 2011. **146**(10): p. 1118.
19. Esposito, C., et al., *Laparoscopic versus open appendectomy in children: a retrospective comparative study of 2,332 cases.* World J Surg, 2007. **31**(4): p. 750-5.
20. Lintula, H., H. Kokki, and K. Vanamo, *Single-blind randomized clinical trial of laparoscopic versus open appendicectomy in children.* British Journal of Surgery, 2001. **88**(4): p. 510-514.

21. Rice-Townsend, S., et al., *Hospital readmission after management of appendicitis at freestanding children's hospitals: contemporary trends and financial implications.* J Pediatr Surg, 2012. **47**(6): p. 1170-6.
22. Oyetunji, T.A., et al., *Pediatric negative appendectomy rate: trend, predictors, and differentials.* J Surg Res, 2012. **173**(1): p. 16-20.
23. Coyne, I. and P. Gallagher, *Participation in communication and decision-making: children and young people's experiences in a hospital setting.* J Clin Nurs, 2011. **20**(15-16): p. 2334-43.
24. Lewis, C.C., R.H. Pantell, and L. Sharp, *Increasing patient knowledge, satisfaction, and involvement: randomized trial of a communication intervention.* Pediatrics, 1991. **88**(2): p. 351-8.
25. Lipstein, E.A., W.B. Brinkman, and M.T. Britto, *What is known about parents' treatment decisions? A narrative review of pediatric decision making.* Med Decis Making, 2012. **32**(2): p. 246-58.
26. Pennarola, B.W., et al., *Factors associated with parental activation in pediatric hematopoietic stem cell transplant.* Med Care Res Rev, 2012. **69**(2): p. 194-214.
27. Post, D.M., D.J. Cegala, and W.F. Miser, *The other half of the whole: teaching patients to communicate with physicians.* Fam Med, 2002. **34**(5): p. 344-52.
28. Lunceford, J.K. and M. Davidian, *Stratification and weighting via the propensity score in estimation of causal treatment effects: a comparative study.* Statistics in Medicine, 2004. **23**(19): p. 2937-2960.
29. Robins, J.M., M.A. Hernan, and B. Brumback, *Marginal structural models and causal inference in epidemiology.* Epidemiology, 2000. **11**(5): p. 550-560.
30. Rosenbaum, P.R. and D.B. Rubin, *The Central Role of the Propensity Score in Observational Studies for Causal Effects.* Biometrika, 1983. **70**(1): p. 41-55.
