# Supplementary material for: Demographic and Clinical Characteristics Associated With the Failure of Nonoperative Management of Uncomplicated Appendicitis in Children: Secondary Analysis of a Nonrandomized Clinical Trial
Source: JAMA Netw Open. 2022 May 2;5(5):e229712. doi: 10.1001/jamanetworkopen.2022.9712 (PMC9062687; doi:10.1001/jamanetworkopen.2022.9712)
Supplement: Supplement 2. — Nonauthor Collaborators [file jamanetwopen-e229712-s002.pdf]

Supplemental Online Content: Nonauthor Collaborators

*Indicates required information. Only first name, last name, and suffix will appear in PubMed.

*Group Name(s): Midwest Pediatric Surgery Consortium							
***First Name and Middle Initial(s)**	***Last Name**	***Suffix (eg, Jr, III)**	Academic Degrees	Institution	Location (city, state/province, country)	Role or Contribution, eg, chair, principal investigator	Group (if more than 1 Group listed in the byline) and/or Subgroup (eg, Steering Committee)
Gail	Benser		MD	Nationwide Children's Hospital	Columbus, OH, USA	Member, MWPSC	
Jessica	Kandel		MD	University of Chicago Medicine - Comer Children's Hospital	Chicago, IL, US	Member, MWPSC	
Frederick	Rescorla		MD	Riley Children's Hospital	Indianapolis, IN	Member, MWPSC	
Daniel	von Allmen		MD	Cincinnati Children's Hospital and Medical Center	Cincinnati, OH	Member, MWPSC	
Brad	Warner		MD	St. Louis Children's Hospital	St. Louis, MO	Member, MWPSC	
Cynthia	Downard		MD	University of Louisville School of Medicine	Louisville, KY	Member, MWPSC	
Beth	Rymeski		MD	Cincinnati Children's Hospital and Medical Center	Cincinnati, OH	Member, MWPSC	
Julia	Grabowski		MD	Ann & Robert Lurie Children's Hospital of Chicago	Chicago, IL, US	Member, MWPSC	
Marleta	Reynolds		MD	Ann & Robert Lurie Children's Hospital of Chicago	Chicago, IL, US	Member, MWPSC	
Carley	Lutz		BS	Nationwide Children's Hospital	Columbus, OH, USA	Research Coordinator, MWPSC	
Sarah	Fox		CCRP	University of Michigan	Ann Arbor, MI	Research Coordinator, MWPSC	
