# Supplementary material for: Demographic and Clinical Characteristics Associated With the Failure of Nonoperative Management of Uncomplicated Appendicitis in Children: Secondary Analysis of a Nonrandomized Clinical Trial
Source: JAMA Netw Open. 2022 May 2;5(5):e229712. doi: 10.1001/jamanetworkopen.2022.9712 (PMC9062687; doi:10.1001/jamanetworkopen.2022.9712)
Supplement: Supplement 3. — Data Sharing Statement [file jamanetwopen-e229712-s003.pdf]

Data Sharing Statement

Minneci. Demographic and Clinical Characteristics Associated With the Failure of Nonoperative Management of Uncomplicated Appendicitis in Children. *JAMA Netw Open*. Published May 02, 2022. doi:10.1001/jamanetworkopen.2022.9712

Data
Data available: No
